# Neuron-astrocyte omnidirectional signaling in neurological health and disease

**DOI:** 10.3389/fnmol.2023.1169320

**Published:** 2023-06-08

**Authors:** Dhruba Pathak, Krishnan Sriram

**Affiliations:** Health Effects Laboratory Division, National Institute for Occupational Safety and Health, Centers for Disease Control and Prevention, Morgantown, WV, United States

**Keywords:** Alzheimer’s disease, astrocyte-neuron communication, glutamic acid, Huntington’s disease, neurodegenerative diseases, Parkinson’s disease, synaptic signaling, tripartite synapse

## Abstract

Astrocytes are an abundantly distributed population of glial cells in the central nervous system (CNS) that perform myriad functions in the normal and injured/diseased brain. Astrocytes exhibit heterogeneous phenotypes in response to various insults, a process known as astrocyte reactivity. The accuracy and precision of brain signaling are primarily based on interactions involving neurons, astrocytes, oligodendrocytes, microglia, pericytes, and dendritic cells within the CNS. Astrocytes have emerged as a critical entity within the brain because of their unique role in recycling neurotransmitters, actively modulating the ionic environment, regulating cholesterol and sphingolipid metabolism, and influencing cellular crosstalk in diverse neural injury conditions and neurodegenerative disorders. However, little is known about how an astrocyte functions in synapse formation, axon specification, neuroplasticity, neural homeostasis, neural network activity following dynamic surveillance, and CNS structure in neurological diseases. Interestingly, the tripartite synapse hypothesis came to light to fill some knowledge gaps that constitute an interaction of a subpopulation of astrocytes, neurons, and synapses. This review highlights astrocytes’ role in health and neurological/neurodegenerative diseases arising from the omnidirectional signaling between astrocytes and neurons at the tripartite synapse. The review also recapitulates the disruption of the tripartite synapse with a focus on perturbations of the homeostatic astrocytic function as a key driver to modulate the molecular and physiological processes toward neurodegenerative diseases.

## 1. Introduction

### 1.1. Historical perspective

Astrocytes are classically depicted as distinctive “star-shaped” (Cajal, 1913) cells derived from the neuroectoderm and localized in the brain and spinal cord. However, the current understanding of the morphology of astrocytes is that astrocytes are bushy- or sponge- like cells (Bushong et al., 2004; Verkhratsky and Parpura, 2016; Schiweck et al., 2018). They are one of the most abundant neuroglial cell types (Nedergaard et al., 2003; Reemst et al., 2016; Lee et al., 2022), and play a critical role in the neural injury. Astrocytes undergo hypertrophy, remodeling, atrophy or degeneration in response to injury, disease or infection of the central nervous system (CNS) (Verkhratsky et al., 2017; Escartin et al., 2021). Tracing back the history of glial cells, pathologist Rudolf Ludwig Carl Virchow, in 1856, first coined the term *neuroglia* referring to it as a ‘glue of the brain,’ merely a homogenous connective tissue structure. Later, in the 1870s, utilizing the silver-chromate staining technique, Camillo Golgi visualized the morphology of astrocytes. In 1893, Mihály Lenhossék (a.k.a. Michael von Lenhossék) coined the term “astrocyte” to describe the star-shaped hollow receptacle (astron, star; kytos, hollow receptacle) in the CNS. All these historical perspectives and recent advancements in the field converged to explain the neuron-astrocyte signaling cascade with/without astrogliopathology consisting of molecular entities, a multitude of receptors, channels, and membrane transporters (Verkhratsky and Nedergaard, 2018), many of which are just beginning to be unraveled. It appeared that an extensive understanding and knowledge of astrocytes grew rapidly after the famous neuroscientist Santiago Ramόn *y* Cajal provided distinct structural elucidations and foresight on their potential diverse functions in the late 19th and early 20th century (Somjen, 1988; Allen and Lyons, 2018).

### 1.2. Overview of the pathological features of astrocytes

Astrocytes have an essential role in health and disease (Allen and Lyons, 2018; Baecher-Allan et al., 2018) with presumed autoimmune etiology. They are widely implicated in most, but not all, pathological processes in the CNS, and their specific response state led to their classification into various astrogliopathologies, including reactive astrogliosis, atrophy, remodeling, and astrodegeneration (Verkhratsky and Parpura, 2016; Verkhratsky et al., 2019a,b). These astrocytes can be converted into reactive astrocytes due to injury and disease (O'Callaghan and Sriram, 2005; Sriram and O’Callaghan, 2005; Pathak and Sriram, 2023), but the mechanism of their induction is incompletely understood (Burda et al., 2016; Liddelow et al., 2017). While astrocytes contribute to the pathogenesis of multiple neurological disorders (Wheeler et al., 2020), the heterogeneity of the astrocytes and their regulatory mechanisms have often baffled researchers.

### 1.3. Morphological and functional heterogeneity of the astrocytes

Astrocytes have not only emerged as a critical entity within the brain because of their unique regulatory role in recycling the neurotransmitters, actively modulating the ionic environment, regulating cholesterol and sphingolipid metabolism, but also in influencing signaling crosstalk in various neurodegenerative disorders (Corkrum et al., 2020; Lezmy et al., 2021; Wang et al., 2021; Iskusnykh et al., 2022). Several studies have also highlighted the fundamental functions of astrocytes that include synaptogenesis, neurovascular coupling, neuronal bioenergetics, protein and waste clearance, sodium (Na^+^) and potassium (K^+^) balance, pH balance, and maintenance of redox status (Bélanger et al., 2011; Xie et al., 2013; Khakh and Sofroniew, 2015; Hillen et al., 2018; Siracusa et al., 2019; Lezmy et al., 2021; Hulshof et al., 2022; Salman et al., 2022). However, little is known about how astrocytes guide synapse formation, synaptic plasticity, CNS homeostasis, and neural network activity through active surveillance and alteration of CNS structure or function (Allen and Lyons, 2018; Perez-Catalan et al., 2021; Allen et al., 2022; Koizumi, 2022).

Astrocytes are armored with thousands of processes that may interact with all cell types of the CNS (Allen and Lyons, 2018). The accuracy and precision of brain signaling are primarily based on the interactions between neurons and various glial cells, including astroglia, oligodendroglia, microglia, and radial glia within the CNS, as well as glial cells like Schwann cells and satellite cells within the peripheral nervous system (see Table 1). For example, a single astrocyte can simultaneously interrelate with nearly 2 million synapses (Fields et al., 2014; Hulshof et al., 2022). Based on the anatomic locations and cellular morphologies, astrocytes have been commonly categorized into protoplasmic and fibrous types (Sofroniew and Vinters, 2010; Lee et al., 2022). Arguably, based on evolutionary trails, morphological appearance, and preponderance, the authors extended their classification of astrocytes to include their presence in vertebrate and invertebrate species and emphasized the need to revisit the patterning and redifferentiation of radial astrocytes (like cerebellar Bergmann glia, retinal Müller glia, cerebellar velate astrocytes, and hypothalamic/spinal tanycytes), ependymal cells, pituicytes, proto-astrocytes (found in roundworms), packet glia and giant glia (found in the leech), perivascular and marginal astrocytes, interlaminar astrocytes, polarized astrocytes, Gomori-positive astrocytes, surface-associated astrocytes, and varicose projections astrocytes in humans, toward a better investigation of the diversity of astrocytes (Verkhratsky et al., 2017; Prevot et al., 2018; Verkhratsky and Nedergaard, 2018 see Table 2). Nonetheless, protoplasmic astrocytes exhibit highly branched structures with webbed feet that are distributed throughout the gray matter, and the mature protoplasmic astrocytes are known to form an elaborative dense ramification of fine processes, resulting in a ‘spongiform’ morphology (Bushong et al., 2004; Sofroniew and Vinters, 2010). They not only envelope the synapses but also wrap around the blood vessels, thereby maintaining the blood–brain barrier’s (BBB) structural integrity. On the other hand, fibrous astrocytes have straight and long fiber-like processes and are abundant in white matter. They are generally associated with the nodes of Ranvier, which are small interspersed unmyelinated regions along the length of an axon that help conduct a nerve impulse (Miller and Raff, 1984; Kimelberg and Nedergaard, 2010; Khakh and Sofroniew, 2015; Lezmy et al., 2021). Both protoplasmic and fibrous astrocytes interact with the distal processes of neighboring astrocytes through gap junctions (Sofroniew and Vinters, 2010). Specifically, the distributed astrocytes in the white and gray matter of the brain and spinal cord are projected as the key homeostatic cells (Verkhratsky et al., 2017). Thus, the simple, supportive role attributed to an astrocyte, as an entity of homogenous cells with similar functions throughout the brain, soon became obsolete once new knowledge about astrocyte’s structural, functional, and physiological diversity came to light (Khakh and Sofroniew, 2015). Further, it highlighted that astrocytic signaling could be differentially regulated within the astrocyte sub-compartments (Khakh and Sofroniew, 2015).

**Table 1:** Types of neuroglial cells.

Cell type	Localization	Physiological function	Reference(s)
Astroglia	CNS (Most abundant and diverse type of glial cell)	Neurotransmitter uptake, Regulation of glutamate, Ion regulation/Potassium buffering, Regulation and maintenance of BBB, Modulation of extracellular matrix, Immunomodulation, Promoting myelination and remyelination, Promote neuronal survival, Neurogenesis, Synaptogenesis, Maintaining extracellular homeostasis.	Araque et al. (1999), Schwarz et al. (2017), Siracusa et al. (2019), and Ohno et al. (2021)
Microglia	CNS (Resident immune cell)	Immune Surveillance, Neuronal defense, Response to injury or disease, Neurogenesis, Control and synaptic density and connectivity, Control of excitotoxicity.	Oberheim et al. (2012), Colonna and Butovsky (2017), and Chavarría et al. (2022)
Oligodendroglia	CNS	Myelination and remyelination, Maintenance of myelin sheath, Surround axons and provide axonal support, Increase the speed of nerve impulses, Metabolic support, Trophic support.	Bradl and Lassmann (2010) and Oberheim et al. (2012)
Radial glia	CNS (Predominantly occurs in the developing CNS)	Precursor cells that can generate other cells including glial cells like oligodendrocytes and astrocytes, Serve as scaffolds to support neuronal migration.	Bentivoglio and Mazzarello (1999) and Campbell and Götz (2002)
Schwann cells	PNS	Formation and maintenance of myelin sheath on axons of peripheral neurons.	Verkhratsky et al. (2017)
Satellite cells	PNS	Support and maintenance of peripheral neurons.	Verkhratsky et al. (2017)

BBB, blood–brain barrier; CNS, central nervous system; CSF, cerebrospinal fluid; PNS, peripheral nervous system.

**Table 2:** Types of astroglial cells.

Cell type	Localization	Physiological function	Reference(s)
Protoplasmic astrocyte	Gray matter- Cerebral cortex, Hippocampus, Cerebellum, Spinal cord	Neurotransmitter uptake, Potassium buffering, Glutamate uptake, Volume regulation, Synapse formation and maintenance, Learning, Memory, Motor coordination.	Araque et al. (1999), Anderson and Swanson (2000), Bushong et al. (2004), Emsley and Macklis (2006), Mishima and Hirase (2010), Sofroniew and Vinters (2010), Oberheim et al. (2012), Khakh and Sofroniew (2015), Adamsky et al. (2018), Siracusa et al. (2019), and Lee et al. (2022)
Fibrous astrocyte	White matter- Corpus callosum, Internal capsule, Spinal cord, PNS	Maintenance of the BBB, Myelination, Axonal support, Structural and metabolic support to axons, Repair of damaged axons, Glia limitans, Formation and maintenance of myelin sheath in the PNS.	Emsley and Macklis (2006), Sofroniew and Vinters (2010), Bélanger et al. (2011), Oberheim et al. (2012), Verkhratsky and Nedergaard (2018), Augusto-Oliveira et al. (2020), and Lee et al. (2022)
Surface associated astrocyte	Posterior prefrontal and amygdaloid cortex	Bidirectional signaling between the brain, pial arteries, and CSF in the subarachnoid space, Transport between the brain, pial arteries, and CSF in the subarachnoid space.	Feig and Haberly (2011) and Verkhratsky and Nedergaard (2018)
Velate astrocyte	Olfactory bulb and granular layer of the cerebellum	Form a sheath around granular neurons.	Emsley and Macklis (2006), Oberheim et al. (2012), and Verkhratsky and Nedergaard (2018)
Gomori astrocyte	Hypothalamus, hippocampus	Iron (heme) transport to neurons, Glucose transport, Promote neurite development, Degradation of toxic lipo-peroxides, Metabolism of neurotransmitters.	Schipper (1991) and Verkhratsky and Nedergaard (2018)
Perivascular and Marginal astrocyte	Close to pia mater for forming endfeet with blood vessels	Establish the pial and perivascular glia limitans barrier.	Emsley and Macklis (2006), Oberheim et al. (2012), and Verkhratsky and Nedergaard (2018)
Pituicyte	Neurohypophysis (Posterior pituitary)	Ion regulation (potassium and glutamate reuptake during secretory activity), Surround axonal endings, Form ‘synaptoid’ contact with neurosecretory axons, which are conduits for glial–neuronal signaling in the posterior pituitary, Storage and release of hormones, Regulation of neurosecretion through the release of taurine, Regulate vascular permeability, Promote permeable (fenestrated) endothelial fate, Block impermeable (tight junction) endothelial fate.	Wittkowski (1998) and Anbalagan et al. (2018)
Ependymocytes (Ependymal cells)	CNS (Specialized cells that line the ventricular walls of the brain and the central canal of the spinal cord)	Maintenance of the structural and functional integrity of fluid-filled cavities (ventricles), Production, circulation, and reabsorption of CSF, Prevention of the entry of toxicants, Repair and regeneration.	Emsley and Macklis (2006), Oberheim et al. (2012), Verkhratsky et al. (2017), and Verkhratsky and Nedergaard (2018)
Bergmann Glia	CNS (Specialized, unipolar glial cells located predominantly in the Purkinje and molecular layer of the cerebellum)	Information processing, Maintenance of structural integrity and synaptic connections, Controlling membrane potential, Regulation of ion and neurotransmitter concentration.	Grosche et al. (1999), Wang et al. (2012), and Leung and Li (2018)
Muller glia	Unique to the retina	Maintain structural integrity of retina, Glucose metabolism, Neurotransmitter uptake, Retinal homeostasis.	Oberheim et al. (2012), Fernández-Sánchez et al. (2015), and Verkhratsky and Nedergaard (2018)

BBB, blood–brain barrier; CNS, central nervous system; CSF, cerebrospinal fluid; GLUT2, glucose transporter 2; PNS, peripheral nervous system.

### 1.4. Quantification of astrocytes: an insight into their composition and regional distribution

The exact ratio of protoplasmic to fibrous astrocytes in the brain must be better characterized. Few studies also acknowledged that it is not a trivial task to label all the cells of the astroglial lineage while understanding the morphological heterogeneity of astrocytes due to the lack of specific universal markers both in *in situ* preparations and *in vivo* in the brain (Verkhratsky and Nedergaard, 2018). Nevertheless, it is known that the astrocyte population and proportion vary within a region and from region to region. Astrocytes roughly account for 20–40% of all glial cells (Verkhratsky and Butt, 2013; Khakh and Sofroniew, 2015). In addition, the proportion of neurons to astrocytes still needs to be fully understood. Quantifying neural (neurons and glia) cells and evaluating their ratios is critical for understanding their regional distribution, cell–cell interactions, as well as developmental, cellular, and structural composition, both under normal and neurological disease states (Morrison and Hof, 1997; Herculano-Houzel, 2009; Herculano-Houzel, 2014; von Bartheld et al., 2016). Further, understanding the threshold and ratio of the morphological transition from resting protoplasmic astrocytes to reactive fibrous astrocytes are important for characterizing the neurophysiological and neuropathological outcomes (Kim et al., 2016). As modern techniques are applied to morphological assessments, more information will likely be unveiled in the forthcoming years. For example, one study compiled all the functional features of astrocytes under one umbrella. It proposed the concept of an “astroglial cradle” that is portrayed as an essential component in synaptogenesis, isolation, and maintenance of synapses, maturation of synapses, the development of synaptic connectivity, and synaptic plasticity (Verkhratsky and Nedergaard, 2014). Despite the variations in the distribution and proportion of astrocytes and neurons across the different brain structures of mammalian species, the interaction between astrocytes and neurons is the fundamental basis for brain function (Herculano-Houzel, 2014).

### 1.5. Strength, opportunities, limitations, and future developments of astrocyte research

Genetically encoded Ca^2+^ indicators, designer receptors exclusively activated by designer drugs, whole-cell patch clamp, super-resolution two photon-imaging, and microscope equipped with resonant scanners and piezoelectric z-drivers are some of the recent advances that are developed in the quest of searching better tools and technology in the neuroscience field that could be instrumental in assessing the astrocyte structural and functional diversity (Khakh and Sofroniew, 2015; Losi et al., 2017). Earlier, electrophysiological research that was centered on the neurons (excitable cells) rather than astrocytes (non-excitable cells) to unravel brain circuits (Adamsky et al., 2018), can now become more interesting if the above-mentioned technologies are utilized in conjunction. Given that astrocytes are electrically silent, they barely deflect by a few millivolts for K^+^ equilibrium potential and exhibit low membrane resistance (Mishima and Hirase, 2010; Khakh and Sofroniew, 2015), but the application of two-photon imaging together with the fluorescently labeled cell and the exploitation of electrophysiology is likely to add new knowledge in the field of heterogeneity of astrocytes. Interestingly, it has been shown by that LTP induction rapidly provoked spatial retreat of glial glutamate transporters, thereby eliciting and enhancing glutamic acid (glutamate; Glu) spillover and consequently leading to NMDA receptor-mediated crosstalk of inter synapses (Henneberger et al., 2020). Thus, with the proper use of improved tools and techniques, all these features previously unappealing to an electrophysiologist at that period in history can be rewarding even though glia was thought of only as supportive cells, electrically silent, and challenging to visualize and assess quickly without fluorescently labeling them. Scientists are actively involved in assessing the full picture of astrocytes in detail to validate the conceptualization such as synaptic cradle and tripartite synapse.

Interestingly, the tripartite synapse hypothesis came to light to fill gaps in the knowledge that constitute a subpopulation of astrocytes, neurons, and synapses. Scientific consensus converged to show that astrocytes not only encapsulate and insulate the synapses but are also actively involved in sensing and modulating synaptic activity (Araque et al., 1999; Adamsky et al., 2018). Delpech et al. (2019) summarized the role of astrocytes in neurological health and neurodegeneration, particularly emphasizing the basic mechanisms of the omnidirectional signaling cascade between astrocytes and neurons. The strength of the present review article is that it illustrates the interaction between neurons and astrocytes during physiological processes, such as learning, memory, and sleep, as well as in psychological, psychiatric, and neurological/neurodegenerative disease states with emphasis on the role of the tripartite synapse. Next, the review discusses the neurotransmitter regulation by astrocytes in the healthy and pathological brain. Characterizing and understanding these interactions and mechanisms can help innovate an effective intervention and therapeutic framework focusing on the transporters and receptors of the astrocytes.

## 2. Physiological and pathophysiological role of astrocytes at the tripartite synapse

**Figure 1:**
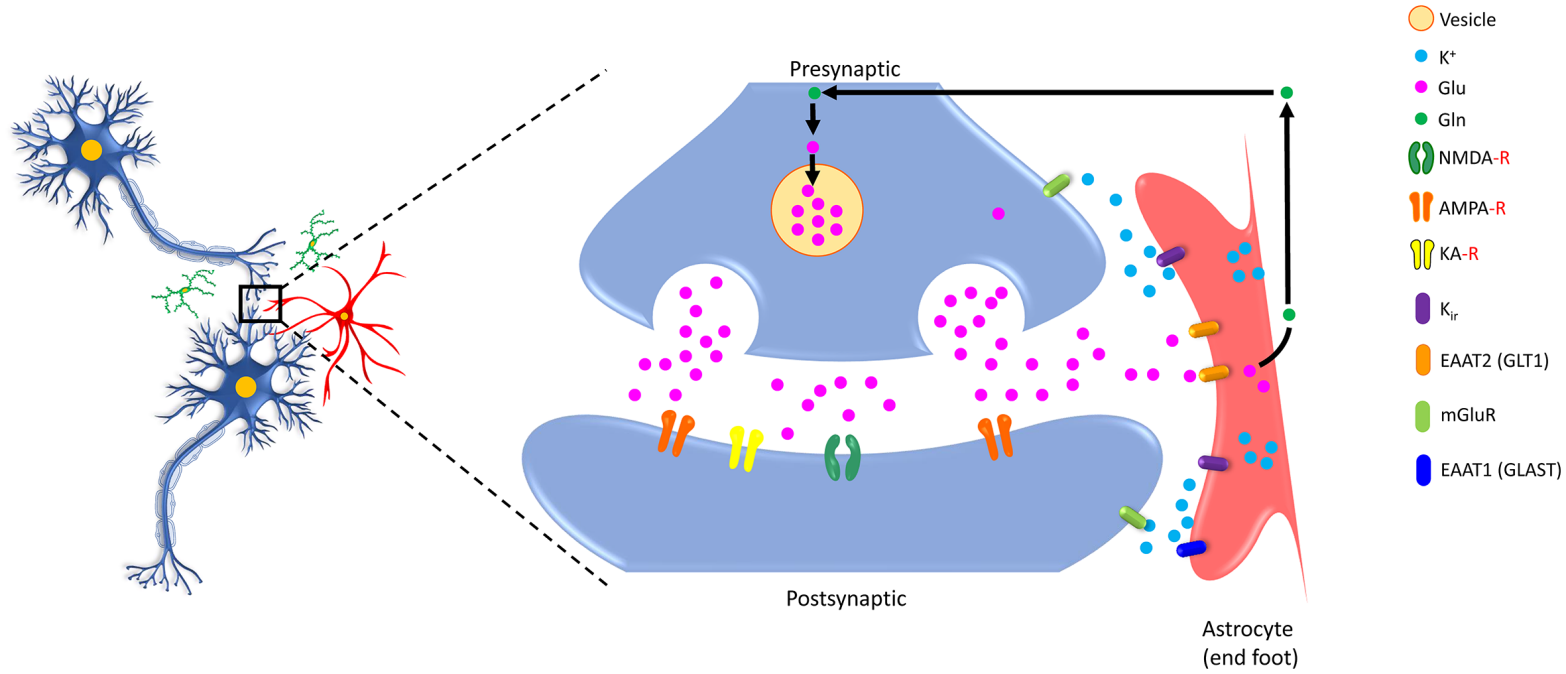
Schematic showing bidirectional communication between the synapses (both presynaptic and postsynaptic) and the astrocytes. Glutamate (Glu) molecules released after the arrival of an action potential at the end of the presynaptic site trigger calcium entry. These transmitters are enclosed in vesicles within the presynaptic site and, upon release, act on their specific receptors (NMDA-R, AMPA-R, KA-R) located on the postsynaptic site, which further elicit downstream signaling cascades. Notably, the presynaptic and postsynaptic sites contain different classes of metabotropic receptor expression sites. Astrocytes are armored with GLT1, GLAST, and K_ir_ that potentially take up excessive Glu and convert it to glutamine (Gln) for recycling back to neurons. K^+^, potassium ion; Glu, glutamic acid (glutamate); Gln, glutamine, NMDA-R, *N*-methyl-d-aspartate receptor; AMPA-R, 2-amino-3-(3-hydroxy-5-methyl-isoxazol-4-yl) propanoic acid receptor; KA-R, kainate receptor; K_ir_, the inwardly rectifying potassium channels; EAAT2 (GLT1), excitatory amino acid transporter 2 (glutamate transporter 1); EAAT1 (GLAST), excitatory amino acid transporter 1 (glutamate aspartate transporter); mGluR, metabotropic glutamate receptors.

Astrocytes are known to make specialized contacts with chemical synapses by enwrapping them, forming the so-called ‘tripartite synapse’ and playing a variety of roles, including regulation of moment-to-moment synaptic transmission and plasticity, synapse formation, maintaining synaptic integrity, and eliminating damaged/degraded synapses (Araque et al., 1999; Pannasch et al., 2011; Hulshof et al., 2022). The concept of tripartite synapse emerged in the late 1990s when the close structural and functional partnership of the peri-synaptic astrocyte processes with neuronal pre- and post-synaptic structures were acknowledged for generating bidirectional communication (Araque et al., 1999; Schwarz et al., 2017; Corkrum et al., 2020). In the classical view of the ‘bipartite synapse,’ information flow from presynaptic to postsynaptic neurons occurs. In contrast, in a tripartite synapse, the astrocytes exchange information with the pre- and post-synaptic neurons during synaptic activity (see Figure 1) and regulate the synaptic neurotransmission (Perea et al., 2009). The formation and functioning of the tripartite synapse are attributed to the interaction between astrocytes and synapses, which is facilitated by cell adhesion and matrix proteins (Hillen et al., 2018).

Astrocytic calcium (Ca^2+^) signaling is extremely fast and localized, supporting the concept that astrocytes are more efficient in regulating a single synapse. These fast signals are mostly independent of neuromodulatory and inositol-1,4,5-triphosphate (IP3) type 2 receptors (IP3R2) but occur exclusively in both astrocyte endfeet and fine processes (Stobart et al., 2018). However, the anatomical basis of such signaling has remained elusive, as previously reported (Araque et al., 1999), due to difficulties in determining the spongiform domain of astrocytes, where most tripartite synapses are localized (Araque et al., 1999; Arizono et al., 2020). Earlier studies reported that astrocytic calcium transients were too slow to generate blood flow increment, and subsequent studies revealed that the most critical calcium transient does evolve in the fine astrocytic process (see Bazargani and Attwell, 2016). In contrast to the neuronal electrical activity, astrocytic calcium activity is far slower and this fluctuating calcium oscillations are mainly determined by features such as IP3R2 signaling complex reverberating interaction between soma and each astrocytic processes, inability to synchronize with other astrocytes, independent of neuronal firing, ability to get modulated by cAMP protein kinase A signaling, and augmentation of energy deprivation and epileptiform activity (Ujita et al., 2017). All these evidence suggest that calcium wave generation may differ within the astrocytic compartments and is likely to be influenced by several parameters as discussed before. Previously, peri-synaptic Schwann cells and synaptically-linked astrocytes were considered integral modulatory elements of tripartite synapse in the peripheral nervous system (Araque et al., 1999). In general, it was proposed that neurotransmitter release from the neuronal synapse activates receptors in astrocytes that increase intracellular Ca^2+^ concentrations, which in turn can spread Ca^2+^ waves to the neighboring astrocytes (astrocyte-astrocyte communication), resulting in the release of gliotransmitters to influence adjacent neuronal activity. Astrocytes also express both Synaptobrevin II (SYB2; VAMP2) and Cellubrevin (CEB; VAMP3) as functional non-overlapping vesicular SNARE proteins mediating Glu and neuropeptide Y (NPY) secretion, respectively, with an antagonistic effect. This in turn regulates the number of readily releasable vesicles, as well as the release probability of fast Glu dynamics at the tripartite synapse, which influences neuron-astrocyte crosstalk within the presynaptic and postsynaptic sites (Schwarz et al., 2017).

Major branches of astrocytes exhibited morphological enlargements due to the activity of local Ca^2+^. However, the basis of Ca^2+^ signaling in the spongiform domain of astrocytes is incompletely understood, even though there is a substantial understanding that astrocytic processes meet thousands of neuronal synapses for regulation (Arizono et al., 2020). Although electron microscopy techniques were able to highlight some of the morphological features of the tripartite synapse in chemically-fixed brain tissues, most of the dendritic spines, neuropils, and extracellular space are better accessible using 3-dimensional Stimulated Emission Depletion (3D-STED) microscopy in unfixed living brain tissues (Tønnesen et al., 2018; Arizono et al., 2020).

Astrocytes have also been reported to express a wide variety of Na^+^ channels, and the intracellular Na^+^ signaling in astrocytes is closely associated with some of their functional characteristic features (McNeill et al., 2021). In a recent study, it has been shown that a chemical or mechanical stimulation can elicit neuronal activity thereby triggering Na^+^ fluxes, which further generate spatio-temporally organized rapid alteration in the cytosolic Na^+^, suggesting a key role for astroglia in establishing the homeostatic response (Verkhratsky and Rose, 2020). Previous cell imaging studies have also shown dynamic changes in intracellular Na(+) concentration {Na[(+)]i} may be ideally positioned for rapid coordination of signaling between neuronal activity and glial “homeostatic” Na (+)-dependent transporters (Rose and Verkhratsky, 2016). Characterization of the role of astroglia in ion balance and equilibrium is still warranted for advancing the field in the light of novel technological developments such as the use of genetically-controlled or membrane-targeted chemical indicators compatible with advanced microscopy techniques like fluorescence-lifetime imaging microscopy (Rose and Verkhratsky, 2016).

In addition to Na^+^ regulation, astrocytes are also known to intensely modulate K^+^ by recruiting a variety of voltage-dependent and voltage-independent channels (McNeill et al., 2021). Astrocytes are linked by gap junctions adding a new dimension in ionic regulation, which permits the redistribution of the accumulated K^+^ from one cell to another soon after the occurrence of intense K^+^ accumulation during extensive neuronal activity (Siracusa et al., 2019). Astroglial gap junction-mediated buffering is still a less understood and sometimes debatable topic of spatial K^+^ buffering in the brain. This is mainly due to the lack of blockers specific to the astrocytic gap junction (Wallraff et al., 2006). Astrocytes interact with neighboring astrocytes through gap junctions (Maragakis and Rothstein, 2006), *via* Connexin-43 protein (also known as gap junction protein alpha 1; GJA1/CX43), a process known to form intercellular syncytial communication. Interestingly, the lack of astroglia CX43 resulted in a transient endothelial activation, an interrupted immune recruitment, along with the development of a specific humoral autoimmune, suggesting their greater role at the gliovascular interface (Boulay et al., 2016). Like CX43, another gap junction protein, Connexin-30 protein (CX30), is also known to mediate the extensive network organization of astrocytes, but their role in cellular physiology remains unknown (Pannasch et al., 2011). To unravel the specific role of astroglial gap junctions in K^+^ buffering, another laboratory obtained the mice with coupling-deficient astrocytes by crossing CX43- and CX30- deficient mice, whereby they observed that the greater extent of K^+^ clearance capacity was conserved in mice with coupling-deficient astrocytes suggesting gap junction-dependent processes (Wallraff et al., 2006). Moreover, these gap junctional networks are thought to modulate the effectiveness of a synaptic population by coordinating the release of gliotransmitters such as Glu, endocannabinoids, adenosine triphosphate (ATP), or dextro-serine (D-Ser), which target the synaptic receptors (Letellier et al., 2016). Thus, CX30 and CX43 form gap junctions that facilitate K^+^ removal and clearance of extracellular Glu.

One of the fundamental features of astrocytes is to take up most of the synaptically-released Glu, which in turn helps in neuronal function and prevents Glu excitotoxicity (Mahmoud et al., 2019). Several studies also reached a similar consensus that Glu has beneficial effects at low levels; however, it can lead to cell death at high extracellular concentrations through excessive activation of Glu receptors, a process referred to as excitotoxicity (Rao and Weiss, 2004; Rudy et al., 2015). At tripartite synapses, the molecular mechanisms that underlie the intracellular regulation of Glu transport have yet to be fully resolved, but it appears that Glu and ion homeostasis are the key components in the development of pathophysiology at the tripartite synapse (Mahmoud et al., 2019; Lee et al., 2022). These astrocytic cells undergo pathophysiological changes, propelling disease progression and altering synaptic transmission. The question of whether fixed categories of astrocytes exist was better explained after identifying the limitations of binary divisions of reactive astrocytes into neurotoxic vs. neuroprotective (A1-*vs*-A2), good vs. bad, and after proposing the guidelines to abrogate the devastating actions of reactive astrocytes and enhance defensive functions (Escartin et al., 2021). Recent studies have shown that any adverse change in the functional or structural nature of the astrocytes directly influences the activity of neurons, consequently leading to neurodevelopmental and neurological disorders (Liu et al., 2021; Perez-Catalan et al., 2021). Thus, stimulus-specific cellular response, cell–cell interactions, and concise nomenclature for astrocytes could assist in glia-to-neuron conversion therapies (Lee et al., 2022).

Astrocyte dysfunction is found in numerous diseases including, Alzheimer’s disease (AD), amyotrophic lateral sclerosis (ALS), Huntington’s disease (HD), and major neuropsychiatric disorders (Lee et al., 2022). A pathological feature of progressive AD is the loss of synapses, which correlates with cognitive decline (Hulshof et al., 2022). In general, tripartite synapses can be a pivot for neurodegenerative diseases, including ALS (Broadhead et al., 2022), AD & PD (Blanco-Suárez et al., 2017). Selective loss of tripartite synapses in the post-mortem spinal cord of patients with ALS is reported (Broadhead et al., 2022). Similarly, loss of postsynaptic structure is observed in the ALS mouse model. Ohno et al. (2021) studied potassium inwardly-rectifying channel subfamily J member 10 (KCNJ10; also known as Kir_4.1_) channels in astrocytes, which mediate spatial K^+^ buffering and clear excess extracellular K^+^ from the tripartite synapses (Ohno et al., 2021). They determined that inhibition of Kir_4.1_ channels elevates K^+^ and Glu levels at the synapse and facilitates brain-derived neurotrophic factor (BDNF) expression in astrocytes, essential for causing excitability in neurons and reducing plasticity and connectivity (Ohno et al., 2021). Likewise, Kir_4.1_ channels are also attenuated in epileptic disorders and Huntington’s disease (HD) but are enhanced in major depressive disorder and neuropathic pain (Khakh and Sofroniew, 2015; Kinboshi et al., 2017; Ohno et al., 2021).

One study compiled the report of several investigations. It highlighted the physiological function and several molecular pathways of astroglia in the association of Na^+^ ion involvement, which includes Na^+^ activated Na_x_ channels, Na^+^-dependent glutamate transporters EAAT1/SLC1A6, and EAAT2/SLC1A2, Cystine/glutamate antiporter Sxc–consisting of xCT/SCL7A11 and 4F2hc/SLC3A2 proteins, Na^+^-dependent GABA transporter GAT3/SLC6A11, Na^+^-dependent glutamine transporters, Glycine Na^+^-dependent glycine transporters GlyT1/SLC6A9, Na^+^-dependent concentrative nucleoside transporters CNT2/SLC28A2 and CNT3/SLC28A3, Na^+^-K^+^ pump (NKA), Na^+^-K^+^-Cl^−^ co-transporter 1 NKCC1/SLC12A2, Na^+^-K^+^-Cl^−^ co-transporter 1 NKCC1/SLC12A2, Na^+^-H^+^ exchanger NHE1/SLC9A1, Na^+^-HCO_3_^−^ transporter NBCe1/SLC4A4, Na^+^-Ca^2+^ exchanger NCX1/SLC8A1, NCX2/SLC8A2 and NCX3/SLC8A3 (Verkhratsky et al., 2017). Any of the above-mentioned astrocyte-based molecular pathways and their perturbation in disease states are not trivial to exclude while studying the omnidirectional neuron-astrocyte signaling cascade.

Interestingly, recent studies have shown that extracellular Glu can stimulate Ca^2+^ release from the astrocytic intracellular stores, further triggering Glu release from astrocytes to the adjacent neurons *via* an exocytotic mechanism (Mahmoud et al., 2019). Astrocytic Ca^2+^ oscillations can promote arachidonic acid accumulation, neuronal synchrony, synaptic crosstalk, gliotransmitter release, induction and progression of the inflammatory state, and alteration of the homoeostatic function. The homeostatic changes include alteration of K^+^ uptake, buffering of ions, and uptake of excitatory neurotransmitters (Araque et al., 1999; Schwarz et al., 2017; Siracusa et al., 2019). To demonstrate the contribution of glutamatergic input in astrocytic Ca^2+^ signaling and neuronal modulation, the glutamate-receptor antagonists, 6-nitro-7-sulfamoyl-benz(f) quinoxalone-2,3 dione (NBQX) and d-2-amino-7-phosphoheptanoic acid (AP7) were used to block glial Ca^2+^ waves that resulted in modulation of synaptic activity. Similarly, the GABA-receptor antagonists, strychnine and bicuculline, have also been effectively used to demonstrate astroglia-induced neuronal/synaptic modulation.

The neuron-centric view projected in the past shadowed the function of astrocytes as a key driver in the pathogenesis of neurological disorders. Still, increasing evidence demonstrates that the concept of astrocytopathies is warranted due to several features such as disruptions of normal astrocyte functions, astrodegeneration, or maladaptive astrogliosis toward the development of neurological diseases (Pekny et al., 2016; Verkhratsky et al., 2019a,b; see Table 3). While the astrocytes are predominantly involved in maintaining the neural tissue’s homeostasis, the failure of the astroglial defense system facilitates the generation of impaired synaptic transmission, neurodegeneration, and death of neurons (Verkhratsky et al., 2019a,b). Thus, it is equally important to investigate the omnidirectional signaling cascade in the light of both neuron-astrocyte viewpoints during astrocytopathies.

**Table 3:** Types of astroglial pathology.

Type	Pathological feature and outcome	Expressed markers	Reference(s)
Reactive astrogliosis	Cell proliferation,	↑ GFAP	O'Callaghan and Sriram (2005), Wilhelmsson et al. (2006), Argaw et al. (2009), Sofroniew and Vinters (2010), Nash et al. (2011), Argaw et al. (2012), Pekny and Pekna (2014), Rocha et al. (2015), Verkhratsky et al. (2016), Verkhratsky et al. (2017), Siracusa et al. (2019), Verkhratsky et al. (2019a), Verkhratsky et al. (2019b), Verkhratsky et al. (2021a), Verkhratsky et al. (2021b), Bozic et al. (2021), and Pathak and Sriram, 2023
	Hypertrophy,	↑ S100B
	Tissue scarring,	↑ VIM
	Inflammation,	↑ NES
	Oxidative stress,	↑ AQP4
	Altered gene expression,	↑ PCNA
	Protein accumulation,	↑ IL1B
	Modification of extracellular matrix,	↑ IL6
	Altered synapse formation	↑ TNFA
		↑ NOS2
		↑ VEGFA
		↑ VCAM1
		↑ MMP9
		↑ CLU
		↑ COL4A1
		↑ CSPG
		↑ NO
Astrocyte atrophy	Decreased size/volume/mass,	↓ GFAP	Verkhratsky et al. (2016), Verkhratsky et al. (2017), Verkhratsky et al. (2019a), Verkhratsky et al. (2019b), Verkhratsky et al. (2021a), Verkhratsky et al. (2021b), and Preman et al. (2021)
	Loss of processes,	↓ S100B
	Downregulation of cellular growth and survival genes,	↓ VIM
	Oxidative stress,	↓ AQP4
	Inflammation,	↓ NO
	Decreased support to neurons,	↓ CLU
	Disruption of the blood–brain barrier	
Astrocyte remodeling	Changes in cell proliferation,	↑ GFAP	Sun et al. (2010), Sun and Jakobs (2012), Verkhratsky et al. (2016), Verkhratsky et al. (2017), Verkhratsky et al. (2019a), Verkhratsky et al. (2019b), Verkhratsky et al. (2021a), and Verkhratsky et al. (2021b)
	Changes in cell migration,	↑ S100B
	Tissue scarring,	↑ VIM
	Inflammation,	↑ AQP4
	Oxidative stress,	↑ IL6
	Altered gene expression,	↑ TNFA
	Protein accumulation,	↑ NOS2
	Modification of the extracellular matrix,	↓ PCNA
	Altered communication with neurons	↓ CLU
		↓ MMP9
Astrocyte degeneration	Decreased size/volume/mass,	↓ GFAP	Verkhratsky et al. (2016), Verkhratsky et al. (2017), Verkhratsky et al. (2019a), Verkhratsky et al. (2019b), Verkhratsky et al. (2021a), and Verkhratsky et al. (2021b)
	Loss of processes,	↓S100B
	Disruption of the blood–brain barrier,	↓VIM
	Oxidative stress,	↓ AQP4
	Inflammation,	↓ PCNA
	Reduction or loss of neural support,	↓ CLU
	Structural changes in the brain,	↓ IL6	
	Formation of vacuoles,	↓ MMP9			Altered distribution of neurotransmitters		

Reactive astrogliosis: Response of astrocytes to injury or damage in the central nervous system. Characterized by the activation and proliferation of astrocytes in response to a stimulus, such as a brain injury, inflammation, or toxicants. Activation leads to changes in the shape, size, and function of astrocytes, which can have positive and negative effects on the nervous system. For example, reactive astrogliosis can help maintain the structural integrity of the brain and support the repair and survival of neurons during injury or disease by releasing trophic growth factors and forming a biomechanical barrier (glial scar) around dying neural cells. On the other hand, it also contributes to the progression of neuronal damage and/or neurodegeneration by releasing proinflammatory mediators. The glial scar can deter the regenerative capability of other neural cells and impair the restoration of normal neural function. Astrocyte atrophy: The shrinking or degeneration of astrocytes. Astrocyte atrophy reduces support, nourishment, and protection afforded to neurons. This can occur due to conditions that affect the health of the nervous system, including aging, traumatic injury, or neuronal neurodegeneration, as seen in AD and PD. Astrocyte remodeling: The change in shape, size, and function of astrocytes in response to various stimuli, including injury, disease, or altered microenvironment. Occurs due to genetic or epigenetic factors, or in response to toxins, inflammation, and oxidative stress. Astrocyte remodeling can have positive and negative effects on the health of the nervous system. For example, astrocyte remodeling can support the survival of neurons and help to maintain the structural integrity of the brain. On the other hand, astrocyte remodeling can contribute to the progression of neural injury and neurodegeneration due to reduced support and increased vulnerability of neurons to oxidative stress and inflammation-mediated damage. Astrocyte degeneration: Refers to the loss of normal structure and function of astrocytes. Astrocyte degeneration can be induced/caused by various factors, including injury, disease, aging, or toxicant exposure. Astrocyte degeneration can have a significant negative impact on the CNS. For example, astrocyte degeneration can disrupt the normal functioning of the BBB, which regulates the transport of molecules between the blood and the brain. This can lead to increased levels of oxidative stress and inflammation, eventually contributing to the death of neural cells. Besides, astrocyte degeneration can also disrupt the normal functioning of astrocytes as supportive cells. For example, astrocytes are important for maintaining the structural integrity of the brain and providing support to neurons. When astrocytes degenerate, this support may be lost, leading to a loss of normal neural function. ↑, Increase; ↓, Decrease; AQP4, Aquaporin 4; CLU, Clusterin; COL4A1, Collagen type IV alpha 1 chain; CSPG, Chondroitin sulfate proteoglycans; GFAP, Glial fibrillary acidic protein; IL1B, Interleukin 1 beta; IL6, Interleukin 6; MMP9, Matrix metalloproteinase 9; NES, Nestin; NO, Nitric oxide; NOS2, Nitric oxide synthase 2; PCNA, Proliferating cell nuclear antigen; S100B, S100 calcium-binding protein B; TNFA, Tumor necrosis factor alpha; VCAM1, Vascular cell adhesion molecule 1; VEGFA, Vascular endothelial growth factor A.

## 3. Physiological and pathophysiological regulation of neurotransmitters by astrocytes

**Figure 2:**
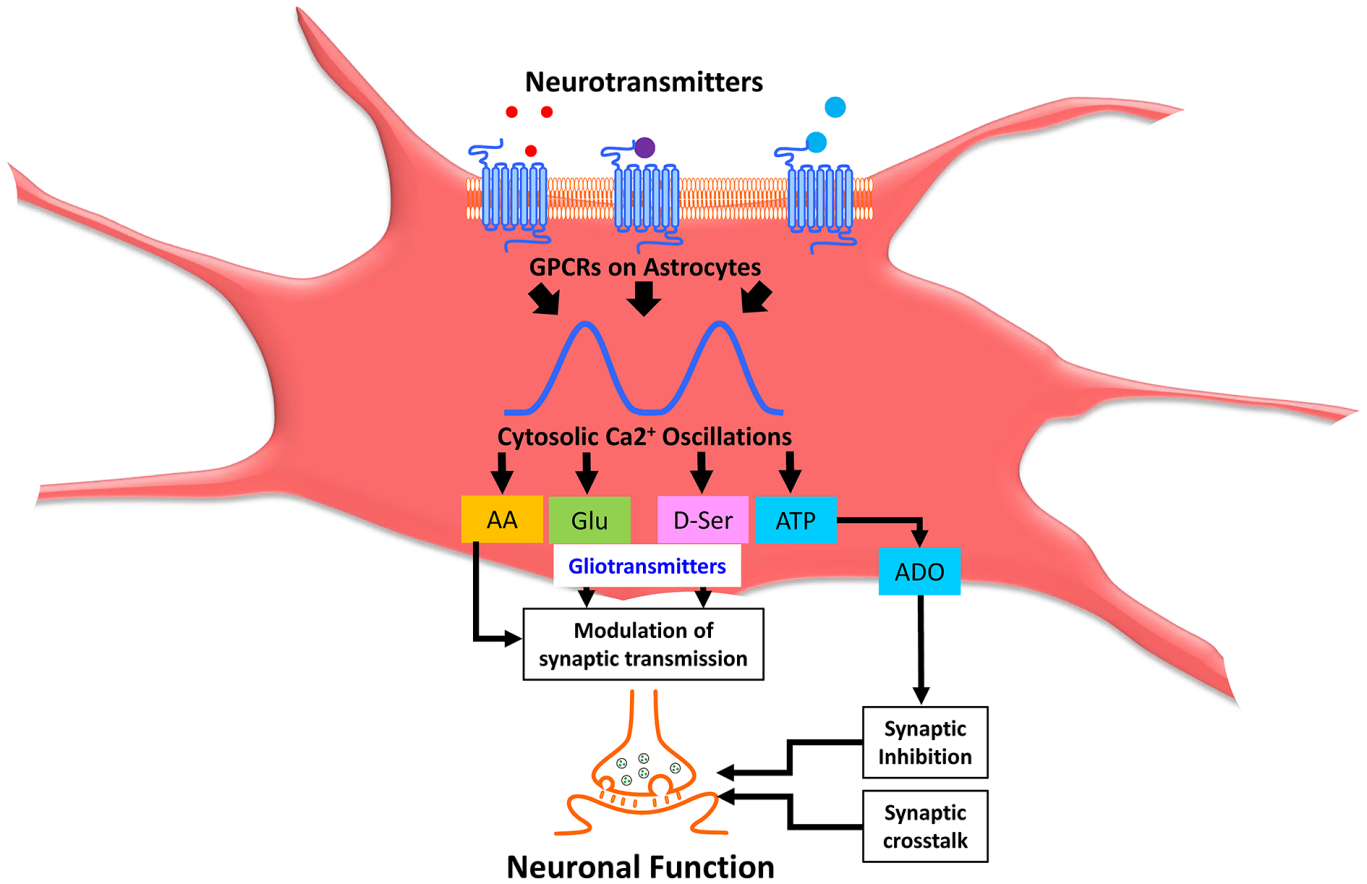
Astrocytic calcium oscillations modulate synaptic neurotransmission through release of gliotransmitters. Neurotransmitters cause activation of various astrocytic receptors leading to cytosolic Ca^2+^ oscillations and subsequent release of a plethora of signaling molecules collectively called gliotransmitters, which constitutes AA, Glu, D-Ser, ATP, ADO, etc. Gliotransmitters, including AA, Glu, and D-Ser, further modulate synaptic transmission. ATP is converted to ADO through a sequential series of enzymatic catalysis, which causes synaptic inhibition. Collectively, synaptic crosstalk and modulation of synaptic transmission alter the neuronal function in the brain. Ca^2+^, calcium; AA, arachidonic acid; Glu, glutamic acid (glutamate); D-Ser, d-dextro-serine; ATP, adenosine triphosphate. ADO, adenosine.

In the healthy CNS, astrocytes are crucial in regulating neurotransmitters vital for the proper functioning of neural circuits. Astrocytes modulate neuronal activity by releasing gliotransmitters since they are not electrically excitable like neurons but capable of employing fluctuations in cytosolic ions as a substrate for exhibiting excitability (Augusto-Oliveira et al., 2020). Synaptic activation of astrocytes leads to the generation of Ca^2+^ signals (see Figure 2), which release chemical transmitters from these astrocytes called gliotransmitters (Halassa et al., 2009). The exocytotic release of the chemical transmitter is one of the mechanisms by which the soluble *N*-ethyl-maleimide-sensitive factor-attachment protein receptors (SNARE) complex between vesicles and the target protein is formed. SNARE complex in astrocytes is a four-helix-bundle protein consisting mainly of Synaptosomal-Associated Protein 23 (SNAP23), Syntaxin 4 (STX4), Vesicle-associated membrane protein 2 (VAMP2; also known as Synaptobrevin 2/SYB2), and Vesicle-associated membrane protein 3 (VAMP2; also known as Cellubrevin/CEB) that regulate synaptic vesicle exocytosis. The astroglial SNARE complex assembly forces the two membranes to fuse tightly with each other to regulate the timing of neurotransmitter release and initiate synaptic transmission (Sutton et al., 1998; Südhof and Rothman, 2009; Vadisiute et al., 2022). This is achieved when vesicular and target membrane localized SNARE proteins zipper up into an alpha-helical bundle that merges both membranes generating the force for fusion (Südhof and Rothman, 2009). When VAMP2 is removed, it reduces fast Ca^2+^-triggered synaptic fusion events by >100- fold and is incompatible with survival (Schoch et al., 2001), pointing out that even small disruptions in the vesicular release have a profound effect in suppressing Glu neurotransmission. In one study, it has been shown that genetically inhibiting the release of gliotransmitter attenuated the accumulation of sleep pressure, and prevented cognitive impairment linked with sleep loss (Halassa et al., 2009). Interestingly, when astrocytes were injected with the light chain of the neurotoxin Botulinum B to selectively cleave VAMP, it resulted in the inhibition of the astrocyte-induced Glu response in neurons (Araque et al., 2000). Despite this understanding, the function of SNARE proteins in astrocytes are still not well established and needs further research (Vadisiute et al., 2022). The signaling molecules that glia release are ATP (Halassa et al., 2009; Koizumi, 2022), lactic acid (Suzuki et al., 2011), glutamic acid, as well as the non-synaptic release of acetylcholine (ACh) & prostaglandin E2 (PGE2; Araque et al., 1999), Glycine (Gly), gamma amino butyric acid (GABA; Benarroch, 2011), and D-Ser (Henneberger et al., 2010).

It is reported that chondroitin sulfate proteoglycans (CSPGs), extracellular matrix proteins produced by mature astrocytes, are also inhibitory for experience-dependent plasticity (Stevens, 2008), indicating that molecules other than gliotransmitters can also be released from astrocytes. It is now increasingly known that astrocytic Glu modulates synaptic transmission (Woo et al., 2012). In contrast, the concentration of Gly at synapses is controlled by two high-affinity sodium (Na^+^) and chloride (Cl^−^) dependent transporters, called solute carrier family 6 (neurotransmitter transporter, glycine), member 9 (SLC6A9; also known as GLYT1) and solute carrier family 6 (neurotransmitter transporter, glycine), member 5 (SLC6A5; also known as GLYT2). GLYT1 is located primarily on astrocytes, and GLYT2 is at presynaptic glycinergic terminals (Benarroch, 2011). Benarroch (2011) highlighted that co-localization and co-release of GABA and Gly mainly occur in the inhibitory terminals within the spinal cord and the brainstem.

Most of the studies on astrocytes were conducted on isolated single cells by patching a single astrocyte to modulate its activity or by uncaging Ca^2+^ in a single astrocyte (Adamsky et al., 2018). It has been shown that modulation of intracellular Ca^2+^ levels in single-cell cultured astrocytes by microinjection (Araque et al., 1999) of the Ca^2+^ chelator BAPTA [1,2-bis(2-amino phenoxy) ethane-*N, N, N9, N9*-tetraacetic acid] or by photolysis of the UV sensitive Ca^2+^-cage (Araque et al., 1999), or with o-Nitrophenyl-Ethylene glycol tetraacetic acid (NP-EGTA), influences the spontaneous neurotransmitter release from the nerve terminals (Araque et al., 1999). Further, mapping astrocytic Ca^2+^ signals in nodes and dendritic spines by 3D-STED microscopy revealed that they are closely associated with individual synapses (Arizono et al., 2020). Together, these above-mentioned ionic signals are tightly linked with the neuronal activity generating a wide array of responses, including activation of plasmalemmal homeostatic transporters, processing, and secretion of a wide variety of neurotransmitters precursors, neuromodulators, cytokines, metabolic substrate, trophic factors and metabolic substrates (Augusto-Oliveira et al., 2020).

Astrocytes immediately respond to any form of CNS insult, exhibiting morphological and functional changes characterized by hypertrophy, enhanced expression of the glial fibrillary acidic protein (GFAP), the release of inflammatory mediators, and the synthesis of neurotrophic factors (Abbracchio and Ceruti, 2006). Such a phenotype is termed ‘reactive astrogliosis.’ In the pathological brain, for example, it has been shown that astrocytic stretch injury can elicit secretion of vasoactive molecules like endothelin 1 (EDN1) and prostaglandin-like compounds like isoprostanes (Hoffman et al., 2000; Ostrow et al., 2011; Burda et al., 2016). Astrocyte stretch injury can also lead to the induction of inositol triphosphate signaling and alter sensitivity to extracellular Glu and inflammatory cytokines (Rzigalinski et al., 1997; Ralay Ranaivo et al., 2011; Burda et al., 2016). In the absence of astroglial Connexin-30 (also known as Gap Junction Protein Beta 6; GJB6) and connexin-43 (also known as Gap Junction Protein Alpha 1; GJA1), it has been shown that astrocyte-dependent Glu recycling and buffering of K^+^ at the synapse are impaired (Perez-Catalan et al., 2021). This culminates in increased excitability at hippocampal *Cornu Ammonis* (CA1) Schaffer collateral synapses (Perez-Catalan et al., 2021) due to enhanced expression of the α-amino-3-hydroxy-5-methyl-4-isoxazole propionic acid receptor (AMPA receptor/AMPA-R). Regulation of the extracellular space between the synapse and the peri-synaptic astrocyte processes (PAPs) governs the diffusion of signaling molecules, including Glu (Hillen et al., 2018). Spontaneous glutamatergic plumes overlapped with a reduced density of glutamate transporter 1 (GLT1)-positive astrocyte processes which were mimicked in wild-type animals by interfering with Glu clearance, thus resulting in the rise of the basal Glu level toward neurological disorders and spreading depolarization (Parker et al., 2021). Massive activation of extra-synaptic *N*-methyl-D-aspartate receptor (NMDA) receptors occurs due to excessive Glu release, which will immediately trigger a shutdown of mitochondrial function leading to severe energy deficiency that cannot be compensated by glycolysis (Bading, 2017). Once the machinery for ATP synthesis is damaged, the cells are at a high risk of death, which could be one of the plausible causes for dysfunctional communication across the tripartite synapse.

## 4. Interaction of astrocytes and neurons

### 4.1. The influence of astrocytes on neuronal activity is regulated by the excitability of neurons, cleaving excitatory neurotransmitters from the synaptic and extra-synaptic spaces and regulating synaptic strength

Neurons and glia are separated by narrow fluid-filled extracellular spaces approximately 20 nm wide. The Glu-gated ion channels participate both in excitatory synaptic transmission, as well as non-synaptic communication within the CNS. However, when an impulse is generated from excitatory or inhibitory neurons and is targeted to the astrocytes, K^+^ is released into the intercellular spaces, depolarizing the glia. This phenomenon is termed non-synaptic signaling. Electron microscopy has provided sufficient insight that there are no gap junctions between neurons and astrocytes. Nevertheless, a low resistance pathway between astrocytes exists, and such extraordinary gap junctional connections are remarkable features of astrocytes. Strikingly, other contacts between neurons and astrocytes could still occur during development and injury. Astrocytes and neurons crosstalk with each other during synaptogenesis, synapse elimination, and synaptic structural plasticity through various secreted and contact-dependent signals (Stevens, 2008).

One of the significant features of the astrocytes is their expression of channels and transporters, which regulates the extracellular concentration of neurotransmitters by modulating the uptake and release of neuroactive agents (Anderson and Swanson, 2000; Woo et al., 2012; Boddum et al., 2016) in response to the network activity. The initial step in this mechanistic regulation is to take excessive Glu from overtly excited neuronal networks, transform it into glutamine (Gln), and finally release Gln into the extracellular space so that the presynaptic terminals can convert it back to Glu (Danbolt, 2001). Both astrocytes and neurons release Glu upon activation. While the neuronal Glu mediates fast synaptic transmission through Ca^2+^-dependent exocytosis, the mechanism of slow synaptic signals mediated *via* astrocytic Glu release still needs to be fully understood. It is thought to be primarily related to modulation, which is highly debated (Woo et al., 2012). This is because, currently, there are no sensitive methods to detect the Glu release from individual astrocytes.

The rapid removal of Glu from the extracellular spaces is essential for neurons’ survival and normal functioning, and astrocytes play a crucial role in its uptake. Glu uptake *via* an astrocyte Na^+^-dependent system with a high affinity for Glu is the primary Glu uptake mechanism in the CNS (Anderson and Swanson, 2000). Within the astrocytes, a portion of the Glu is converted to Gln. A large part is transported back to neurons *via* the excitatory amino acid transporters (EAAT), which belong to the solute carrier 1 (SLC1) family of transporters. Of the five subtypes of EAAT/SLC1s known, glutamate/aspartate transporter 1 (GLAST1/EAAT1/SLC1A3) and glutamate transporter 1 (GLT1/EAAT2/SLC1A2) are the high-affinity Glu transporters that help to cycle Glu back to neurons and facilitate resynthesis and reuse of Glu. In addition to Glu, glial-cell-released ATP and D-Ser mediate robust synaptic actions (Haydon and Carmignoto, 2006), as evidenced by subcellular imaging of Ca^2+^ signals.

The physiological functions of gamma-aminobutyric acid (GABA) released from astrocytes are unclear. Boddum et al., 2016 reported a novel form of inhibitory GABA receptor whose function is mainly dependent on the astrocytic GABA transporter 3 (GAT3/SLC6A11) activity that leads to astrocytic Na^+^ accumulation and thereby causing increments in astrocytic Ca^2+^ *via* Na^+^/Ca^2+^ exchange, and eventually resulting in the release of astrocytic ATP/Adenosine (ADO). It is now well accepted that such an extracellular rise of ADO can efficiently inhibit Glu release machinery acting *via* presynaptic ADO receptors (Boddum et al., 2016). Nonetheless, Na^+^ channels are scarcely distributed on astrocytes and contribute little to its conductance. On the other hand, astrocytes are high in K^+^ concentrations and exhibit low permeability to other ions.

Astrocytes eliminate functionally impaired and excessive synapses (Lee et al., 2021), pointing toward a role in mediating neuronal activity-dependent elimination of excitatory synapses. Stimulation of synaptic NMDARs has been shown to elicit NMDAR-induced responses acting mainly *via* nuclear Ca^2+^ signaling cascade, which is thought to afford a ‘neuroprotective shield.’ In contrast, stimulation of the extra-synaptic NMDARs promotes neural cell death (Hardingham and Bading, 2010). This double-edged role is thought to be due to the activation of distinct genomic signatures and effects on intracellular signaling cascades. The perturbations in the balance between synaptic and extra-synaptic NMDA-R activity can contribute to neuronal impairment and is likely a common event in the etiopathogenesis of many neurodegenerative disorders (Hardingham and Bading, 2010).

Kruyer et al. (2022) studied the effects of opioid use on astrocytes and determined two forms of plasticity relying on astroglial GLT1. They showed that in one form of plasticity, astroglia exhibited increased synapse proximity, and this occurred selectively at the D2-Dopamine receptor (DRD2)-expressing dendrites. At the same time, changes in GLT1 were not neuron subtype-specific. In the other form of plasticity, increased morphological proximity to synapses occurred in one sub-population, while increases in extra-synaptic GLT1 expression occurred in another sub-population of cells (Kruyer et al., 2022). Another study elegantly showed that increasing neuronal activity, in the absence of learning, directly produced memory impairment. On the other hand, an astrocyte-mediated increase in neuronal activity during learning-associated tasks, was shown to enhance memory (Adamsky et al., 2018). Astrocyte activation is essential for synaptic plasticity and sufficient to cause induction of NMDA-dependent *de novo* synaptic potentiation in the hippocampus. This synaptic potentiation mediated by astrocytes persists even after cessation of astrocytic activation (Adamsky et al., 2018). It is now well accepted that activation of extra-synaptic NMDA receptors results in neurodegeneration and cell death mediated through several biochemical and pathological processes such as mitochondrial dysfunction, nuclear accumulation of class IIA histone deacetylases (encoded by HDAC2), disruption of cyclic adenosine monophosphate-responsive element-binding protein (CREB), the elevation of extracellular Glu concentrations in the different synaptic sites, and loss of integrity of neuronal structures and connectivity (Bading, 2017).

Among the different forms of plasticity reported, high frequency stimulation (HFS)-evoked long-term depression (LTD) is one of the highly discussed topics in the field. On the one hand, the postsynaptic depolarization combined with HFS is thought to lead to the dendritic release of signaling molecules that directly or indirectly modulate astrocyte signaling (Nagai et al., 2019). On the other hand, the physiological function of extra-synaptic NMDARs is not entirely understood. However, Glu spillover is thought to contribute to their activation and long-term depression (Massey et al., 2004; Hardingham and Bading, 2010). In these studies, the authors determined that the induction of long-term depression (LTD) is enhanced by blocking Glu uptake that then activates extrasynaptically located *N*-methyl d-aspartate receptor subtype 2B (NR2B/GRIN2B) receptors, whereas the de-potentiation phenomenon requires activation of *N*-methyl d-aspartate receptor subtype 2A (NR2A/GRIN2A) receptors. Selective inhibition of GRIN2B receptors was sufficient to prevent the LTD induction phenomenon, which elucidated that this subtype of NMDA receptor has functions in synaptic plasticity. Furthermore, LTD requires both synaptic and extra-synaptic receptors (Papouin et al., 2012), while synaptic and extra-synaptic NMDA-Rs are gated by distinct endogenous co-agonists, D-Ser and Gly, respectively (Armada-Moreira et al., 2020). These electrophysiological experiments performed on the CA1 region of the hippocampus provided a fundamental understanding of the functional disparities between synaptic and extra-synaptic NMDARs in brain physiology and pathological processes.

Neuronal networks are highly efficient in rapidly altering their structural and functional status, changes that are termed plasticity. Such features are critical for shaping neural circuits during neurodevelopment (Perez-Catalan et al., 2021). Notably, such features are equally important in memory formation, behavior, and neurological disease. Astrocyte development in the CNS occurs at late embryogenesis, which correlates with the onset of sensory-evoked activity (Perez-Catalan et al., 2021), thus making the research field of neural plasticity more exciting (Perez-Catalan et al., 2021).

The involvement of astrocytes in long-term potentiation (LTP) induction has always remained a topic of debate. The neuronally-released D-Ser is the one that regulates NMDA-R activity, and D-Ser is abundant in the protoplasmic astrocytes close to the NMDA-Rs, but not in neurons. Thus, NMDA-Rs are essential for the binding of D-Ser or Gly, at the glycine modulatory site (GMS) to be functionally effective. Clamping the internal Ca^2+^ in individual CA1 astrocytes has been shown to block LTP in excitatory synapses by reducing the occupancy of the NMDAR co-agonist sites (Henneberger et al., 2010; Ohno et al., 2021). This evidence showed that the Ca^2+^-dependent release of D-Ser from astrocytes regulates the NMDA-R-dependent plasticity of neighboring excitatory synapses (Henneberger et al., 2010; Ohno et al., 2021). Furthermore, they showed that LTP blockade was reversible by exogenous D-Ser or Gly. At the same time, depleting D-Ser or disrupting exocytosis in an individual astrocyte was sufficient to block local LTP (Henneberger et al., 2010).

Astrocytes can modulate synaptic strength by increasing spontaneous excitatory post-synaptic currents (Nedergaard et al., 2003), where astrocytic Ca^2+^ is associated with brief modulation of calcium strength. The fundamental mechanism that regulates synaptic efficacy relies on the depolarization of the astrocytic membranes, activation of astrocytic NMDA receptors, activation of astrocytic L-type Ca^2+^ channels, and enhancement of astrocyte calcium signaling (Letellier et al., 2016). These studies support the notion that astrocytes relay signals to neurons to modulate synaptic strength. During the astrocyte-neuron communication, astrocytes express synaptobrevin II (SYB2/VAMP2) and cellubrevin (SYB3/VAMP3) as functionally non-overlapping vesicular SNARE proteins on glutamatergic vesicles and neuropeptide Y (NPY)-containing large dense-core vesicles (Schwarz et al., 2017). Interestingly, the authors showed that astroglial VAMP3-dependent NPY secretion diminished synaptic signaling. However, VAMP2-dependent Glu release from astrocytes enhanced synaptic signaling. The changes in the synaptic strength are critical. It is suggested that they should be carefully regulated, as uncontrolled synaptic activity between neurons can result in abnormal neuronal activity patterns, loss of synaptic sensitivity, and eventually excitotoxicity (Perez-Catalan et al., 2021).

### 4.2. Role of astrocyte-neuron communication in physiological processes such as learning, memory, and sleep

#### 4.2.1. Astrocytes and learning

Astrocytes exhibit biological properties that influence learning and cognition (Fields et al., 2014). LTP, one of the prime forms of synaptic plasticity, epitomizes the physiological and cellular basis for understanding memory (Petravicz et al., 2014; Allen et al., 2022; Lee et al., 2022). However, the precise cellular mechanism of learning remains obscure. A recent study has shown that neuronal networks are highly efficient in rapidly altering their functional and structural characteristics, a process termed plasticity. Such features shape neural circuits during neurodevelopment (Perez-Catalan et al., 2021).

Suzuki et al. (2011) proposed that synaptic activity associated with learning triggers astrocytic glycogen breakdown. This releases lactic acid mediated *via* monocarboxylate transporter 1 (MCT1/SLC16A1) and monocarboxylate transporter 4 (MCT4/SLC16A4), as demonstrated in the rat hippocampus (Suzuki et al., 2011). The released lactic acid is taken up by monocarboxylate transporter 2 (MCT2/SLC16A7), which triggers the expression of the activity-regulated cytoskeleton-associated protein (ARC) gene and phosphorylation of the nuclear transcription factor *cAMP response element-binding* protein (CREB) and the actin-binding proteins, Cofilins (CFL1 and CFL2). These features contribute to the memory consolidation processes (Bélanger et al., 2011; Suzuki et al., 2011). In this signaling cascade, structural synaptic changes are likely to occur that are essential for long-term memory.

It is now well-accepted that learning is highly dependent on states of arousal, sleep cycle, motivation, attention, and prior history of experiences (Xie et al., 2013; Fields et al., 2014). Interestingly, when large portions of mouse astrocytes were replaced by human astrocytes making it a humanized chimeric mouse, it resulted in enhanced synaptic plasticity and a faster ability to learn (Han et al., 2013; Fields et al., 2014). Astrocytes from various mammalian species share identical features. Still, human astrocytes account for the most complex and largest astrocytes studied (Verkhratsky et al., 2017), making humanized chimeric mouse model more relevant to mimic neurological disease state. Collectively, these findings demonstrate the crucial role of astrocytes in synapse formation, synaptic function, and synaptic plasticity. Perturbation of astrocyte structure or function could thus be determinantal for normal synaptic function, and lead to synaptic dysfunction and neurological disease states.

#### 4.2.2. Astrocytes and memory

Long-term potentiation of synaptic transmission is a useful model for studying the mechanisms of memory (Henneberger et al., 2010; Ohno et al., 2021; Allen et al., 2022). It has been shown that astrocytic glycogen breakdown and associated lactic acid release are essential for influencing higher brain functions, including long-term memory (Suzuki et al., 2011; Armada-Moreira et al., 2020). Inhibition of glycogen phosphorylase interrupts memory consolidation (Bélanger et al., 2011). Further, glycogenolysis and astrocytic lactic acid transporters are crucial in the molecular machinery underlying memory formation (Suzuki et al., 2011).

Several neuronal processes are regulated by astrocytes, such as synapse formation and modulation of synaptic plasticity. Memory dysfunction in autism spectrum disorder (ASD) has been linked to astrocyte abnormalities (Allen et al., 2022). ASD astrocytes play a role in the induction of repetitive behavior and impaired memory through modulation of neuronal network activity and spine density (Allen et al., 2022). Within the range of nanomolar to micromolar concentration, soluble beta-amyloid (Aβ) oligomers impair excitatory synaptic transmission, inhibit LTP, induce loss of dendritic spines, and impair spatial memory in rodents (Crews and Masliah, 2010).

Intriguingly, metabolic compartmentation between astrocytes and neurons provided evidence that the neuro-energetic interactions also act as signaling events in controlling crucial brain functions like memory consolidation (Bélanger et al., 2011).

#### 4.2.3. Astrocytes and sleep

Nature has bestowed the animal kingdom to undergo sleep. Little is known about how animals and humans retrieve memory after they experience the sleep phase. However, previous studies have shown that memory consolidation/retrieval during sleep or rest largely depends on the sharp wave-ripple (SPR-W) complex in the hippocampus-entorhinal cortex areas that result in the transfer of labile memories from the hippocampus to the neocortex (Girardeau et al., 2009). Still, it is now recognized that sleep helps to clear the metabolic, toxic waste in the brain that accumulates because of brain activity. Different patterns of oscillations can assist with this crucial task in the brain. In astrocytes, the mammalian brain-type fatty acid binding protein (FABP7) is widely expressed (Gerstner et al., 2017). Its functions are associated with metabolic, inflammatory, and homeostatic pathways, and its mRNA oscillates in tandem with the sleep–wake cycle (Gerstner et al., 2017). Indeed, Gerstner et al. (2017) have shown that mice and fruit flies deficient in FABP7 or carrying FABP7.T61M missense mutation in the astrocytes exhibited fragmented sleep. This finding is important because it opens the linking pathway of lipid-signaling cascades within astrocytes in sleep regulation. Interestingly, it was reported that during natural sleep or while under anesthesia, the interstitial space increased by 60%, resulting in a prominent rise in the convective exchange of cerebrospinal fluid and interstitial fluid (Xie et al., 2013; Iliff and Simon, 2019), and these convective fluxes of interstitial fluid augmented the rate of peptide, proteins, and oligomers, including Aβ clearance, during sleep.

During sleep deprivation, more ATP is released and metabolized to ADO, which has an anti-depressive effect (Koizumi, 2022). Thus, astrocytes likely play an essential role in the glial purinergic signaling cascade during the pathogenesis of depression. Astrocyte Ca^2+^-evoked ATP release can be converted to ADO at the extracellular site. There is diversity in astrocyte-derived ADO levels between the wake and sleep states or during energy deficiency, which controls the flow of information in white matter and neural circuit formation (Haydon, 2017; Lezmy et al., 2021; Ohno et al., 2021).

Neurons are known to regulate the electrical synaptic transmission of signals, but astrocytes do not communicate with electric impulses. Rather they signal *via* molecules toward spatial integration and long-term temporal regulation in complex cognitive functions. In this context, Bojarskaite et al. (2020) studied the essential role of astrocytic Ca^2+^ signaling in regulating sleep. They found that astrocytic Ca^2+^ signals exhibited distinct features during the sleep–wake cycle. Astrocytic Ca^2+^ signals are reduced during sleep compared to the wakeful state. Genetic ablation of inositol 1,4,5-trisphosphate receptor type 2 (ITPR2), a critical astrocytic Ca^2+^ signaling pathway, has been shown to cause impairment of slow wave sleep, increased slow wave sleep state transitions, increased sleep spindles, microarousals, and aberrant brain rhythms (Bojarskaite et al., 2020). Remarkably, it has been shown that blocking gliotransmission diminished the accumulation of sleep pressure (Halassa et al., 2009), as evidenced by changes in slow wave activity of the electroencephalogram (EEG) during Non-rapid eye movement (NREM) sleep and prevented sleep loss associated with cognitive deficit/dysfunction (Halassa et al., 2009).

### 4.3. Role of astrocyte-neuron communication in psychological and psychiatric disorders such as anxiety, depression, mood disorders, PTSD, mania, and schizophrenia

Astrocyte-neuron communication in psychological and psychiatric disorders has attracted enormous interest in mental health research because little is known about the mechanisms of action. Astrocyte-neuron communication mediators could serve as potential therapeutic targets. However, research in this area is still in the primitive stage to provide a framework for developing new medicinal drugs.

#### 4.3.1. Astrocytes and anxiety

Anxiety disorder is characterized by persistent and excessive worry, fear, and avoidance of perceived threats (Zhao et al., 2021). It has been shown that astrocyte-mediated activation of transforming growth factor-*β*-activated-kinase 1 (TAK1) in the mediobasal hypothalamus (MBH) mitigated anxiety-like behaviors (Zhao et al., 2021). In contrast, suppressing the expression of TAK1 in MBH astrocytes promoted anxiety-like behavior in mice (Zhao et al., 2021). These observations suggest a role for astrocyte signaling in regulating anxiety. A specific population of astrocytes that express the oxytocin receptor (OXTR) has been shown to mediate anxiolytic and positive reinforcement in the central amygdala (Ohno et al., 2021) of rodents treated with the posterior pituitary hormone, oxytocin (Ohno et al., 2021; Wahis et al., 2021). Astrocyte activation mediated by adenosine A1 receptors (ADORA1) has been shown to disrupt memory consolidation and decrease contextual memory but did not cue fear memory which was accompanied by reduced fear-related anxiety behavior (Khanna and Carmena, 2017; Li et al., 2020).

Benzodiazepines enhance GABA neurotransmission, which is anxiolytic. Electrophysiological studies examining the excitability of neurons and the inhibitory effects of GABAergic synaptic neurotransmission in the central nucleus of the amygdala (CeA) of homozygous IL6 transgenic mice showed enhanced excitability of the CeA neurons. These findings suggest a role for astrocyte-derived IL6 in eliciting the exploratory drive and depression-like behavior (Roberts et al., 2019). Astrocytes solitarily express inositol 1,4,5-trisphosphate receptor type 2 (IP3R2/ITPR2), and ITPR2 conditional knockout mice do not display anxiety or depression-like behavior or changes in motor and sensory function (Petravicz et al., 2014).

#### 4.3.2. Astrocytes and depression

Depression and related mood disorders remain among the most significant public health concerns. Depression exhibits various symptoms involving complex dysfunction of neuronal systems, including the monoaminergic neurons (Koizumi, 2022). A recent study showed that synchronized activity of NMDA-Rs and T-type voltage-sensitive calcium channels (T-VSCCs/CACNA1G, CACNA1H, CACNA1I, also known as Ca_V_3.1, Ca_V_3.2, and Ca_V_3.3, respectively) caused rapid bursts of neuronal firing in the lateral brain habenula (LHb), which led to depression-like symptoms in rats (Yang et al., 2018). Yang et al. (2018) also observed that locally blocking the NMDA-R or T-VSCCs in the LHb elicited rapid antidepressant effects. Using the conditional Cre recombinase/locus of x-over, P1 (Cre-*loxP*) system to precisely remove nuclear receptor subfamily 1 group H member 2 (NR1H2/LXRBB) from astrocytes (Li et al., 2021), it was shown that NR1H2/LXRB deletion caused anxiety-like, but not depressive-like, behavior in mice (Li et al., 2021; Lee et al., 2022), thus ruling out the involvement of NR1H2 / LXRB in depressive behavior.

#### 4.3.3. Astrocytes and mood disorders

Mood fluctuations are expected during everyday life events, but severe and persistent mood swings may result in psychological and behavioral distress. Pyramidal neurons in layer V of the medial prefrontal cortex (mPFC) are primarily involved in mood behaviors, where astrocytic NR1H2 (LXRBB) is critical for synaptic transmission and thus is a potential therapeutic target for the treatment of mood disorders and anxiety-like behavior (Li et al., 2021; Lee et al., 2022). There are also increasing reports that abnormal glucocorticoid signaling and altered glial-neuron communication in CNS are essential for emotional response (Horchar and Wohleb, 2019; Madeira et al., 2022). Abnormal functioning of the astrocytes, which contribute to altered glucocorticoid signaling in the brain, is mediated by adenosine receptor subtype A2a (ADORA2A/A2aR), which reinforces the relevance of ADORA2 in mood disorders (Madeira et al., 2022). Using the glucocorticoid, Dexamethasone (DEX) to mimic early life stress and anxiety/mood-like behavior, Madeira et al. (2022) showed that DEX could enhance ATP and Glu release, as well as increase the basal astrocytic Ca^2+^ levels, suggestive of the involvement of astrocytic glucocorticoid signaling in stress and mood-related disorders. In addition, quantification of astrocytes and glial cells revealed that it is reduced in the prefrontal, orbitofrontal area and anterior cingulate, entorhinal and subgenual cortex, and amygdala of the brain investigated from the samples derived from patients with debilitating depression and bipolar disorder (Verkhratsky et al., 2019a,b).

#### 4.3.4. Astrocytes and post-traumatic stress disorder

Post-traumatic stress disorder (PTSD) is the most predominant psychological consequence of exposure to traumatic stimuli related to events like violence, injury, and death. The biological correlates of PTSD include genes, neuroendocrine hormones, and inflammatory markers. The functional correlates include, autonomic risk, resilience, fear learning, anxiety, emotion regulation, and contextual processing (Shalev et al., 2017). However, the underlying neural mechanism of PTSD is not entirely understood. Astrocytes, the homeostatic cells in the CNS, are intimately associated with the pathophysiological outcomes of various mental health conditions, including PTSD (Ji et al., 2023). The nucleotide-binding oligomerization domain leucine-rich repeat and pyrin domain containing protein-3 (NLRP3)-mediated inflammasome (Engin and Engin, 2021) activation in PTSD causes mitochondrial impairment by suppressing ATP synthesis and increasing reactive oxygen species (ROS). Interestingly, stimulation of signal transducer and activator of transcription 3 (STAT3) in astrocytes could reverse the mitochondrial impairment (Engin and Engin, 2021) and thus may be a novel candidate for the pharmacological treatment of PTSD. Another recent study reported that pre-existing diabetes accentuates PTSD-like symptoms but not astrocyte activation in the hippocampus (Ribeiro et al., 2021). These types of studies help understand the association of metabolic disorders with PTSD.

#### 4.3.5. Astrocytes and mania

Mania is a mental and behavioral health condition (Sartorius et al., 2021) characterized by abnormally elevated extreme changes in mood or emotions, arousal, energy, and activity levels (Berrios, 2004). The underlying mechanism of mania is unknown; however, dysfunction of the right prefrontal cortex has been commonly linked (Clark and Sahakian, 2008). Histological assessments have shown diffused white matter alterations in the midline structures (Magioncalda et al., 2016) and a reduced number of glial cells in the brain (Ongür et al., 1998). Proton magnetic resonance spectroscopy studies have demonstrated a significantly higher Gln/Glu ratio in the anterior cingulate cortex and parieto-occipital cortices of patients with bipolar disorders in maniac episodes compared to healthy subjects (Öngür et al., 2008). This line of the investigation indicates that the astrocytes may participate intensely in regulating the Gln/Glu ratio during mania.

#### 4.3.6. Astrocytes and schizophrenia

Schizophrenia is a psychiatric syndrome characterized by hallucinations, delusions, speech problems, reduced motivation, diminished expressiveness, and impaired cognitive executive memory functions. The cellular neurobiology of schizophrenia is not fully understood. In schizophrenia, astrocytes exhibit a variety of features with a marked down-regulation of expression of multiple astroglia-specific molecules, mostly in the deep layers of anterior cingulate gyrus, including deiodinase type II, thrombospondin, glutamine synthetase, plasmalemmal glutamate transporters, and S100β (Verkhratsky et al., 2019a,b).

Various reports from electrophysiological studies revealed that abnormalities in the synchronized oscillatory activity of neurons might be critical for the pathophysiology of schizophrenia. Glu and dopamine (Uno and Coyle, 2019) have been central to studying schizophrenia. Deficient Glu transmission *via* NMDA-Rs has been documented in schizophrenia, suggesting the astrocytic release of D-Ser may contribute to this outcome (Blanco-Suárez et al., 2017). In schizophrenic patients, the synchronization of beta and gamma electrical wave activity is abnormal (Uhlhaas and Singer, 2010), pointing to a crucial role for dysfunctional oscillations in the brain’s rhythm-generating neural networks (Uhlhaas and Singer, 2010). Such a network comprises of GABAergic interneurons and cortico-cortical connections. Their dysfunction results in cognitive deficits and other learning/memory symptoms (Uhlhaas and Singer, 2010). A failure of astrocytic differentiation may result in abnormal glial coverage, delayed maturation, and deficiency of astrocytes, which in parallel, leads to disruption of glutamatergic, potassium, and neuro-modulatory homeostasis, finally culminating in dysregulation of synaptic transmission (Dietz et al., 2020).

### 4.4. Role of astrocyte-neuron communication in neurological/neurodegenerative conditions such as epilepsy, Alzheimer’s, Parkinson’s, Huntington’s, multiple sclerosis, amyotrophic lateral sclerosis/motor neuron diseases

Astrocytes are involved in the neuropathology of neurological and neurodegenerative diseases, primarily due to the imbalance arising from the loss of normal homeostatic function and the gain of toxic function (Phatnani and Maniatis, 2015). Further, the presence of astrocytic cytoplasmic inclusions, protein aggregates like alpha-synuclein (SNCA), Aβ, and Tau in neurons, can also contribute to neuropathology (Li and Haney, 2020) and appears to be one of the critical features in many neurodegenerative diseases. Such astrocytic protein aggregates can compromise the phagocytic function of the astrocytes, dysregulate the astrocytic processes, and render the neurons more vulnerable to adverse factors in their micro-environment, thereby affecting neuronal viability (Phatnani and Maniatis, 2015; Li and Haney, 2020). In addition, the astrocytes can be transformed into different compromised states resulting in atrophy with loss of function and reactive astrogliosis with hypertrophy. These reactive astroglial changes are implicated in senile plaques at the early to moderate stage of AD during mild cognitive impairment and in the late stage of the disease during severe dementia (Verkhratsky et al., 2019a,b).

For decades, studies focused on the synaptic or neuronal level to understand the mechanism of neurological or neurodegenerative disease; however, the role of astrocytes in mammalian behavior, neurological, and neurodegenerative processes remain less understood.

#### 4.4.1. Astrocytes and epilepsy

Epilepsy is a severe neurological disorder characterized by an imbalance in excitatory and inhibitory functions, which arises mainly due to a disturbance in the metabolism and recycling of the neurotransmitter Glu. Evidence accumulates that dysfunctional astrocytes are central players in epilepsy; however, the origin, propagation, and the termination of epileptiform activities remain elusive. Interestingly, GLT1 which is located in astrocytes showed diminished functional activity that eventually decreased Glu clearance and increased epileptogenic foci (Peterson and Binder, 2019; Siracusa et al., 2019). The astrocyte-specific enzyme glutamine synthetase (GS) is known to catalyze the conversion of ammonia and Glu to Gln. In temporal lobe epilepsy, there is a decrease in glutamine synthetase (also known as glutamate-ammonia ligase; GLUL) in epileptic patients, thereby causing hyperexcitability due to the slow pace of Glu-Gln cycling (Steinhäuser et al., 2012). Indeed, GS expression levels or their activity are profoundly deficient in patients with mesial temporal lobe epilepsy (MTLE; van der Hel et al., 2005; Eid et al., 2008). Although GS activity-disrupting mutations have been reported previously in human studies, heterozygote mutations were not clearly linked with seizures or epilepsy (van Gassen et al., 2009). In the mouse model of epilepsy, GS is directly phosphorylated on threonine residue 301 (T301) within the enzyme’s active site by cAMP-dependent protein kinase (PKA) resulting in a dramatic decrease in glutamine synthesis along with enhanced T301 phosphorylation (Huyghe et al., 2019). Furthermore, another study showed that conditional ablation of GS expression selectively in the mouse cortex generated spontaneous seizures (Zhou et al., 2019).

**Figure 3:**
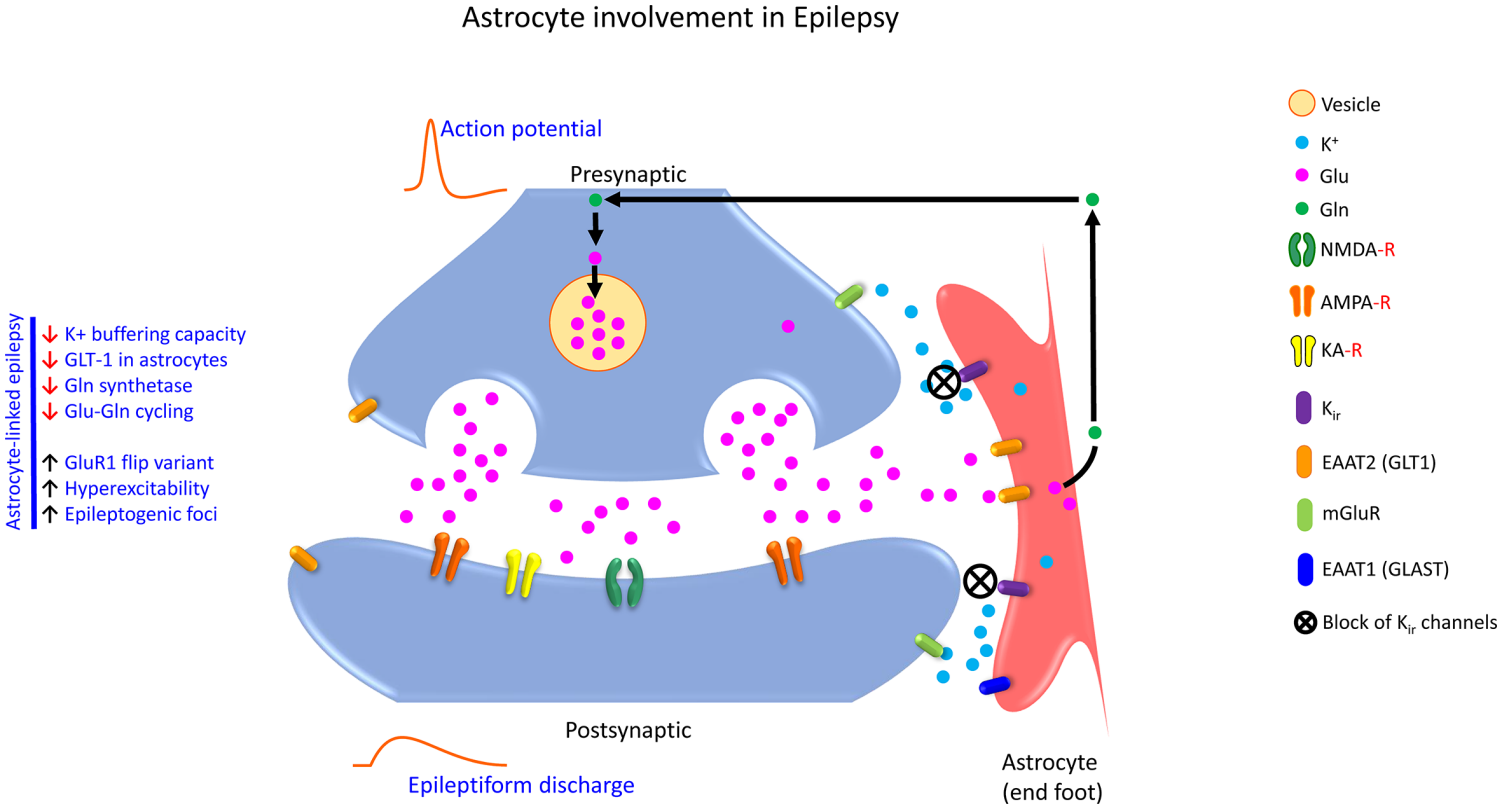
Schematic showing the astrocyte involvement in the tripartite glutamatergic synapse in epilepsy. Accumulation of Glu in the extracellular space upregulates the expression GLT1 and GLAST on the astrocytes, through which Glu is taken up from the synaptic cleft by the astrocyte. GLUL catalyzes the conversion of Glu to Gln. Enhanced Glu release increases epileptogenic foci, GluR1 flip variant, and hyperexcitability. In temporal lobe epilepsy, the Glu-Gln recycling is slowed, which results in hyperexcitability. Similarly, increased expression of GluR1 flip variants in hippocampal astrocytes causes prolonged receptor opening for Na^+^ and Ca^2+^, thus reducing the K^+^ buffering capacity of the astrocytes as a direct block of astroglia K_ir_ channels. The excess Glu can activate extra-synaptic NMDA receptors *via* the excitotoxic pathway causing synaptic loss and, eventually neuronal cell death. To that end, astrocyte-linked epilepsy exhibit features like decreased K^+^ buffering capacity, reduced GLT1 levels, reduced Gln synthetase activity, and perturbation of the Glu-Gln cycling. K^+^, potassium ion; Glu, glutamic acid (glutamate); GLUL, glutamine synthetase; Gln, glutamine; NMDA-R, *N*-methyl-d-aspartate receptor; AMPA-R, 2-amino-3-(3-hydroxy-5-methyl-isoxazol-4-yl) propanoic acid receptor; KA-R, Kainate receptor; K_ir_, the inwardly rectifying potassium channels; EAAT2 (GLT1), excitatory amino acid transporter 2 (glutamate transporter 1); EAAT1 (GLAST), excitatory amino acid transporter 1 (glutamate–aspartate transporter); mGluR, metabotropic glutamate receptors.

It has also been reported that increased expression of AMPAR subunit glutamate receptor 1 (GluR1) flip variants in hippocampal astrocytes cause prolonged receptor opening for Na^+^ and Ca^2+^ ions, thereby reducing the K^+^ buffering capacity of astrocytes as a direct block of astroglial K_ir_ channels (Steinhäuser et al., 2012). Abnormalities of Na^+^/K^+^ ATPase (NKA) activity in epilepsy has been reported (Verkhratsky et al., 2019a,b), and the involvement of protein kinases and phosphatases in regulating such neuronal NKA activity is also evident (Mohan et al., 2019). During the intense neuronal activity, extracellular K^+^ levels increase from 3 mM to 10–12 mM (Armada-Moreira et al., 2020). The astrocytic cellular system is designed to handle such significant increases by facilitating its uptake. Beyond NKA activity, Kir4.1 is one of the major inwardly rectifying K(+) channels expressed mostly in glial cells and instrumental in regulating K^+^ homeostasis (Seifert et al., 2009). Genetic screens and clinical studies suggest that loss-of-function of KCNJ10 encoding Kir4.1 in humans causes EAST (Epilepsy, Ataxia, Sensorineural deafness, and Tubulopathy) or SeSAME (Seizures, Sensorineural deafness, Ataxia, Mental retardation, and Electrolyte imbalance; Bockenhauer et al., 2009; Scholl et al., 2009; Ohno, 2018). In addition, knockout of Kir4.1 in rodent models has been reported to cause severe neurological deficits, including sensorineural deafness, ataxia, epileptiform activities, and early postnatal death (Nwaobi et al., 2016). All support the notion that Kir4.1 dysfunction facilitates neuronal hyperexcitability and is likely to contribute to epilepsy, thus warranting more detailed studies to reveal the role of Kir4.1 in epilepsy. However, blockade of inward rectifying K-channels (K_ir_) depolarizes the neuron, prolongs depolarization, and leads to either epileptiform activity or cellular death (see Figure 3). Moreover, abnormal electrophysiological parameters arising due to the activity of ion channels, receptors, and transporters were noted in astrocytes isolated from patients with mesial temporal lobe epilepsy and associated sclerosis (Verkhratsky et al., 2019a,b).

Besides the functions of Kir4.1, the glia water channel aquoporin-4 (AQP4) is involved in the control of ion and osmohomeostasis and its deletion results in an inflammatory response evidenced by the secretion of different proinflammatory cytokines such as interleukin 1 beta (IL1B), interleukin 6 (IL6), and inducible nitric oxide synthase (NOS2; Pannicke et al., 2010). Analyzing the specimens obtained from patients with pharmaco-resistant temporal lobe epilepsy and epilepsy models showed changes in expression, localization, and function of astroglial K^+^ and AQP4 channels, which resulted in impaired K^+^ buffering (Steinhäuser and Seifert, 2012). When wildtype and AQP4-deficient mice were given a low dose of the chemoconvulsant pentylenetetrazol (PTZ), it was observed that all wildtype mice exhibited seizure activity, whereas most of the AQP4-deficient mice did not produce any seizure activity (Binder et al., 2004). However, at a higher dose of PTZ, both groups showed seizure activity. Still, the latency of generalized tonic–clonic seizures was significantly lower in wildtype mice as compared to AQP4-deficient mice (Binder et al., 2004), providing evidence that glial water channels can modulate excitability and seizure activity. Interestingly, AQP4 knockout mice produced seizures for a longer period and slowed K^+^ kinetics, and the seizure latency was increased when the hippocampal circuit was electrically stimulated (Binder et al., 2006).

To investigate astrocyte uncoupling and the development of chronic mesial temporal lobe epilepsy (MTLE), several studies utilized transgenic mice lacking CX30 and CX43 in astrocytes, the deficiency of which resulted in hyperexcitability (Wallraff et al., 2006), but not spontaneous seizures or abnormal EEG response (Chever et al., 2016). In another mouse model of MTLE, it was observed that astrocyte uncoupling and suppression of K^+^ clearance temporally resulted in neuronal death and the onset of the spontaneous epileptiform activity, indicating this type of uncoupling for the generation of MTLE (Bedner et al., 2015). In addition, continuous telemetric EEG recordings and video monitoring showed increased seizure and interictal spike activity in CX-deficient mice as compared to wildtype mice, without causing a change in the severity of *status epilepticus* (Deshpande et al., 2020). Although several studies have supported the view that CX36 may also be involved in epileptogenesis, there is still an ongoing controversy associated with the alterations of CX36 at different developmental stages of human and animal models of epileptogenesis (Wu et al., 2018). CX43 is increasingly expressed in the hippocampus tissue resected from the patients with MTLE and was proposed for exacerbating generalized seizures in the progression of MTLE (Fonseca et al., 2002). In contrast, a decline in CX43 mRNA levels and unaltered protein levels were also reported in hippocampal tissue from patients with a complex partial seizure disorder (Elisevich et al., 1997).

#### 4.4.2. Astrocytes and Alzheimer’s disease

Astrocytes are an increasingly discussed topic in AD because cellular and molecular changes can result in lower synapse numbers, deficits in eliminating senescent synapses, and causes synaptotoxicity before cognitive decline. Astrocytes easily convert to reactive phenotypes in AD with an altered expression profile and function of healthy astrocytes (Hulshof et al., 2022). Reactive astrocytes are generated in molecularly defined programs involving transcriptional regulation and can undergo molecular, morphological, or functional remodeling in response to disease or insult to the brain (Sriram et al., 2004; O'Callaghan and Sriram, 2005; Sriram and O’Callaghan, 2005). Synaptic loss caused by the release of Aβ_(1–42)_ in AD occurs *via* α7 nicotinic acetylcholine receptors [also known as cholinergic receptor nicotinic alpha 7 subunit/CHRNA7; Talantova et al., 2013]. Aβ engages CHRNA7 receptors to elicit the release of astrocytic Glu, which activates extra-synaptic NMDA-Rs on neurons to mediate synaptic damage. The authors reported that reduced miniature excitatory postsynaptic current (mEPSCs) might be the signature of early synaptic injury due to extra-synaptic NMDA-R-mediated nitric oxide (NO) synthesis, phosphorylation of tau, and caspase-3 activation, resulting in the loss of dendritic spines. However, they also reported that synaptic NMDA-R activation is neuroprotective *in vivo* and *in vitro* (Talantova et al., 2013).

**Figure 4:**
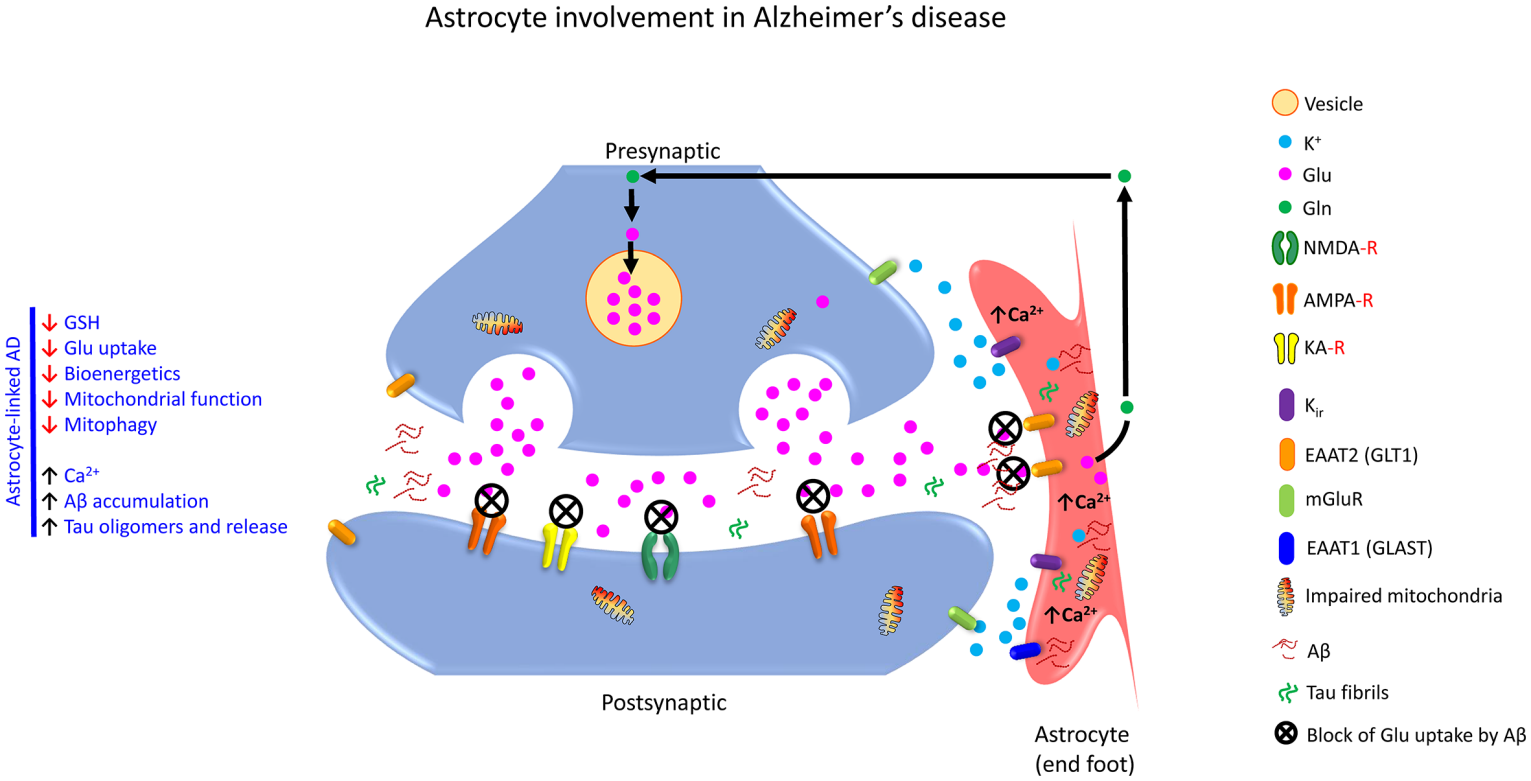
Schematic exhibiting astrocyte involvement in the tripartite glutamatergic synapse in AD. Accumulation of Aβ and Tau fibrils alters astrocyte function (reactive astrogliosis)during synaptic transmission in a tripartite synapse. Aβ fibrils initially originate as the deposition of Aβ-peptides, the cleavage product of amyloid precursor protein (APP), which act to block the astrocytic Glu uptake. In response, astrocytes influence the uptake Ca^2+^, actively promote the accumulation of Aβ and Tau fibrils, as well as release inflammatory mediators. These events result in decreased GSH, Glu uptake, bioenergetics, and mitochondrial function, culminating in neuronal damage/injury/dysfunction. K^+^, potassium ion; Glu, glutamic acid (glutamate); Gln, Glutamine, NMDA-R, *N*-methyl-d-aspartate receptor; AMPA-R,2-amino-3-(3-hydroxy-5-methyl-isoxazol-4-yl) propanoic acid receptor; KA-R, kainate receptor; K_ir_, the inwardly rectifying potassium channels; EAAT2 (GLT1), excitatory amino acid transporter 2 (glutamate transporter 1); EAAT1 (GLAST), excitatory amino acid transporter 1 (glutamate aspartate transporter); mGluR, metabotropic glutamate receptor; Aβ, beta-amyloid fibrils; tau, tau fibrils.

Reactive astrocytes are found surrounding Aβ plaques in the hippocampus of APP/PS1 mice, enwrapping and engulfing the axonal and presynaptic dystrophies to facilitate the elimination of aberrant presynaptic elements (Gomez-Arboledas et al., 2018). Nevertheless, the involvement of astrocytes in binding and degrading Aβ_(1–42)_ is well known (Wyss-Coray et al., 2003). The astrocytes tend to migrate toward sites of neural injury or lesions in response to chemotactic stimuli (for example, monocyte chemoattractant protein 1; MCP1 release); however, they appear to cease migration upon interaction with immobilized Aβ_(1–42)_ (Wyss-Coray et al., 2003). Thus, reduced migration and impaired astroglial clearance of amyloid peptides may likely contribute to the pathogenesis seen in AD (see Figure 4).

To this end, although astrocytes are more resilient than neurons, loss of primary and secondary astrocyte processes has been observed in mouse models of AD (Olabarria et al., 2010; Lee et al., 2022). While signal transducer and activator of transcription 3 (STAT3) are activated in an AD mouse model and human AD, targeted deletion of astrocytic STAT3 in APP/PS1 mice resulted in reduced Aβ and plaque burden (Reichenbach et al., 2019). The authors determined that STAT3 deletion in astrocytes strongly ameliorated spatial learning and memory decline in APP/PSI mice (Reichenbach et al., 2019). Thus, JAK-STAT3 signaling in astrocytes has essential roles in both exacerbation and the mitigation of neurodegeneration (Sriram et al., 2004; Lee et al., 2022).

More recent studies showed that astrocyte-based expression of phagocytic receptors such as C-mer proto-oncogene tyrosine kinase (MERTK) and multiple epidermal growth factor-like domains protein 10 (MEGF10) are down-regulated in AD, resulting in an increase in neuronal damage in one-year-old APP751_sl_ mice (Sanchez-Mico et al., 2021). An earlier study (Shi et al., 2017) determined that increased astrocytic C3 protein expression induced synapse loss at the proximity of amyloid plaques, ultimately triggering neurodegeneration. Several lines of evidence show that ApoE4-mediated astrocytic protein changes have a multitude of damaging effects that include reduced spine density and shorter spine length (Nwabuisi-Heath et al., 2014), increased tau-mediated synaptic loss (Wang et al., 2021), reduced expression of postsynaptic density protein 95 (PSD-95, also known as synapse-associated protein 90; encoded by the gene disks large MAGUK scaffold protein 4; DLG4) and Synapsin (Zhao et al., 2017), impaired Aβ uptake and cholesterol accumulation (Lin et al., 2018), and increased number of non-functional synapses due to decreased phagocytic functions (Chung et al., 2013). Recently, cannabinoid receptor 1 (CNR1) expression was discovered in astrocytes, which also has been shown to take part in long-term plasticity (Arjmand et al., 2022).

**Figure 5:**
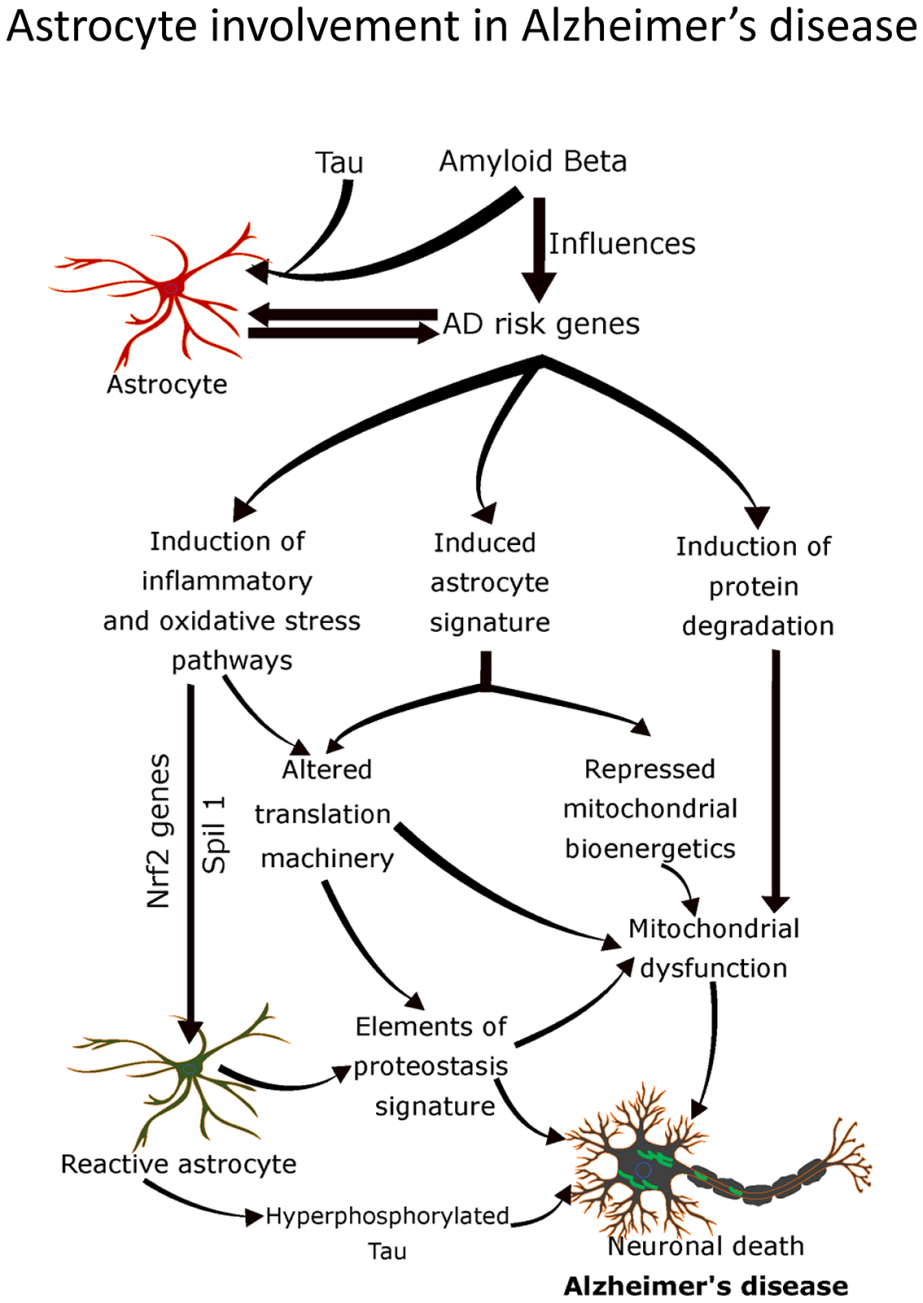
Schematic showing astrocyte involvement in AD. The molecular hallmarks of AD, Aβ fibrils, and Tau fibrils, playing a role in altering the astrocyte function (reactive astrogliosis). Aβ fibrils originate as the deposition of Aβ-peptides, the cleavage products of amyloid precursor protein (APP). AD risk genes have many actions in the signaling cascades including, induction of inflammatory and oxidative stress pathways, induction of astrocyte signature, and induction of protein degradation. This interlinked signaling cascade has broader implications, including altered translation machinery causing mitochondrial dysfunction, elements of proteostasis signature such as phagocytosis, hyperphosphorylation of Tau, synapse loss, and neuronal death. Two genes, Nrf2 and Spil2, have been identified to act on the inflammatory pathways to convert normal astrocytes into a reactive phenotype that causes synaptic loss and eventually neuronal cell death.

Accumulating Aβ, inflammation, and impaired Glu uptake in astrocytes (see Figure 5) can elicit oxidative stress and mitochondrial dysfunction in AD (Khakh and Sofroniew, 2015; Zhao et al., 2017). Superoxide Dismutase 2, Mitochondrial (also known as Manganese-Containing Superoxide Dismutase; SOD2/MnSOD) has been shown to reduce protein nitration, lipid peroxidation, and apoptosis caused by Aβ (Maragakis and Rothstein, 2006).

#### 4.4.3. Astrocytes and Parkinson’s disease

**Figure 6:**
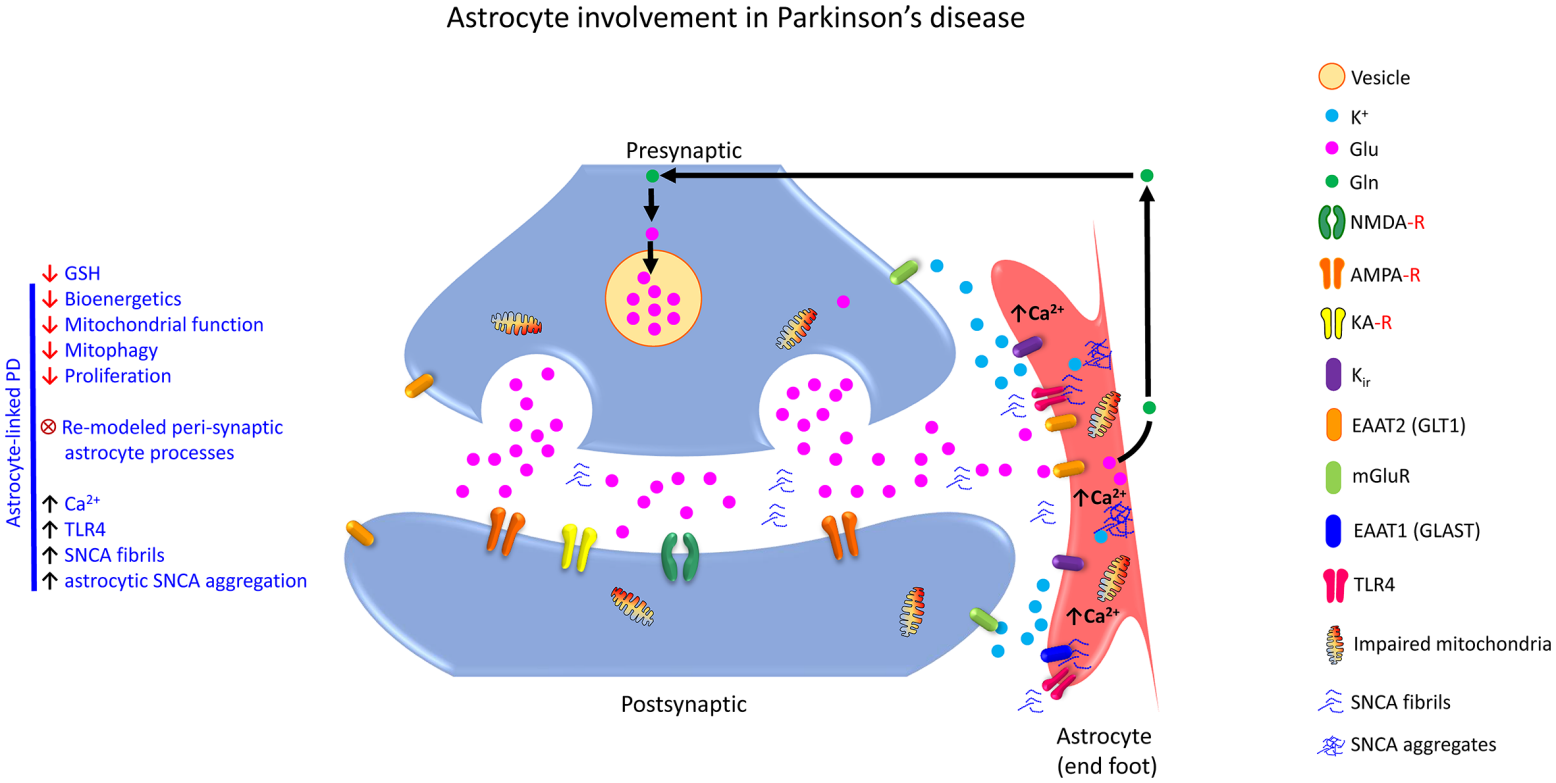
Schematic showing astrocyte involvement in the tripartite glutamatergic synapse in PD. Accumulation of SNCA fibrils and SNCA aggregates alter the astrocyte function (reactive astrogliosis). The mechanism for the progression and initiation of pathogenesis PD includes a decrease in GSH content, bioenergetics, and mitochondrial function. Augmentation of global Ca^2+^ signal, TLR4-like receptor function, SNCA fibrils, and astrocytic SNCA aggregation further act on the signaling pathways to convert astrocytes into reactive phenotypes. In addition, re-modeled peri-synaptic astrocyte processes are also a silent feature of astrocyte-linked PD. This interlinked signaling cascade has broader implications, including mitophagy, mitochondrial dysfunction, proteasome dysfunction, synapse loss, and neuronal death. K^+^, potassium ion, Glu, glutamic acid (glutamate); Gln, glutamine, NMDA-R, *N*-methyl-d-aspartate receptor; AMPA-R, 2-amino-3-(3-hydroxy-5-methyl-isoxazol-4-yl) propanoic acid receptor; KA-R, kainate receptor; K_ir_, the inwardly rectifying potassium channels; EAAT2 (GLT1), excitatory amino acid transporter 2 (glutamate transporter 1); EAAT1 (GLAST), excitatory amino acid transporter 1 (glutamate aspartate transporter); mGluR, metabotropic glutamate receptor; TLR4, toll-like receptor; SNCA, alpha-synuclein.

Parkinson’s disease (PD) is the second most crucial neurodegenerative disorder after AD, which originates due to the loss of dopamine neurons in the substantia nigra pars compacta and abnormal aggregation of misfolded α-synuclein (SNCA) in Lewy bodies (Lee et al., 2022). Contrary to the extensive literature on neurons, astrocyte involvement in dopaminergic signaling and injury is not entirely understood. Dopamine is associated with physiological processes like learning, memory, motor control, and reward, as well as pathological conditions such as PD (Corkrum et al., 2020). The symptoms associated with PD are motor impairments and non-motor outcomes, including cognitive dysfunction. Little is known about the initiation and progression of PD. However, increasing evidence suggests that pro-inflammatory glial response (see Figure 6) contributes to PD pathology (Lee et al., 2022). Membrane-associated gamma-glutamyl transpeptidase (GGT) localized on the surface of astrocytes (Iskusnykh et al., 2022) plays a role in converting extracellular glutathione (GSH) to the dipeptide Cysteine-Glycine (Cys-Gly), and subsequently to Cys and Gly, which are then transported into neurons to serve as precursors for the synthesis of neuronal GSH (Iskusnykh et al., 2022). It was shown that the concentration of GSH decreases in the substantia nigra, during both the ontogenesis and degeneration of dopaminergic neurons in patients with PD (Iskusnykh et al., 2022). In experiments using brain slices, it has been shown that dopamine released at the synapse (Corkrum et al., 2020; Ohno et al., 2021) augments the astrocyte Ca^2+^, which kindles ATP/ADO release and dampens the excitatory synaptic neurotransmission through activation of the presynaptic Adenosine A1 Receptors (Corkrum et al., 2020; Ohno et al., 2021).

Neuronal SNCA can directly translocate to the astrocytes *via* sequential exocytosis and endocytosis to elicit an inflammatory response (Lee et al., 2010; Sorrentino et al., 2019; Chavarría et al., 2022). Specifically, the higher-order SNCA aggregates play a role in microglial activation and astrogliosis (Chavarría et al., 2022), which is mediated through several receptors. Astrocytic SNCA-immunoreactive inclusions have been noted in sporadic PD with neuropathological stage 4 or higher. The first observed pathology was shown in the layers V-VI of the temporal mesocortex, followed by the striatum, and subsequently in thalamic nuclei that project to the cortex (Braak et al., 2007; Ohno et al., 2021). The dopaminergic neurotoxicant, 1-methyl-4-phenyl-1,2,3,6-tetrahydropyridine (MPTP), has been shown to selectively damage striatal dopaminergic nerve terminals and elicit a glia response through rapid tyrosine (Tyr-705) phosphorylation and nuclear translocation of STAT3 in striatal astrocytes (Sriram et al., 2004). Further, deficiency of TNF receptors protected against MPTP-induced striatal dopaminergic neurotoxicity suggestive of a role for TNFA in neurodegeneration (Sriram et al., 2002, 2006a,b).

#### 4.4.4. Astrocytes and Huntington’s disease

**Figure 7:**
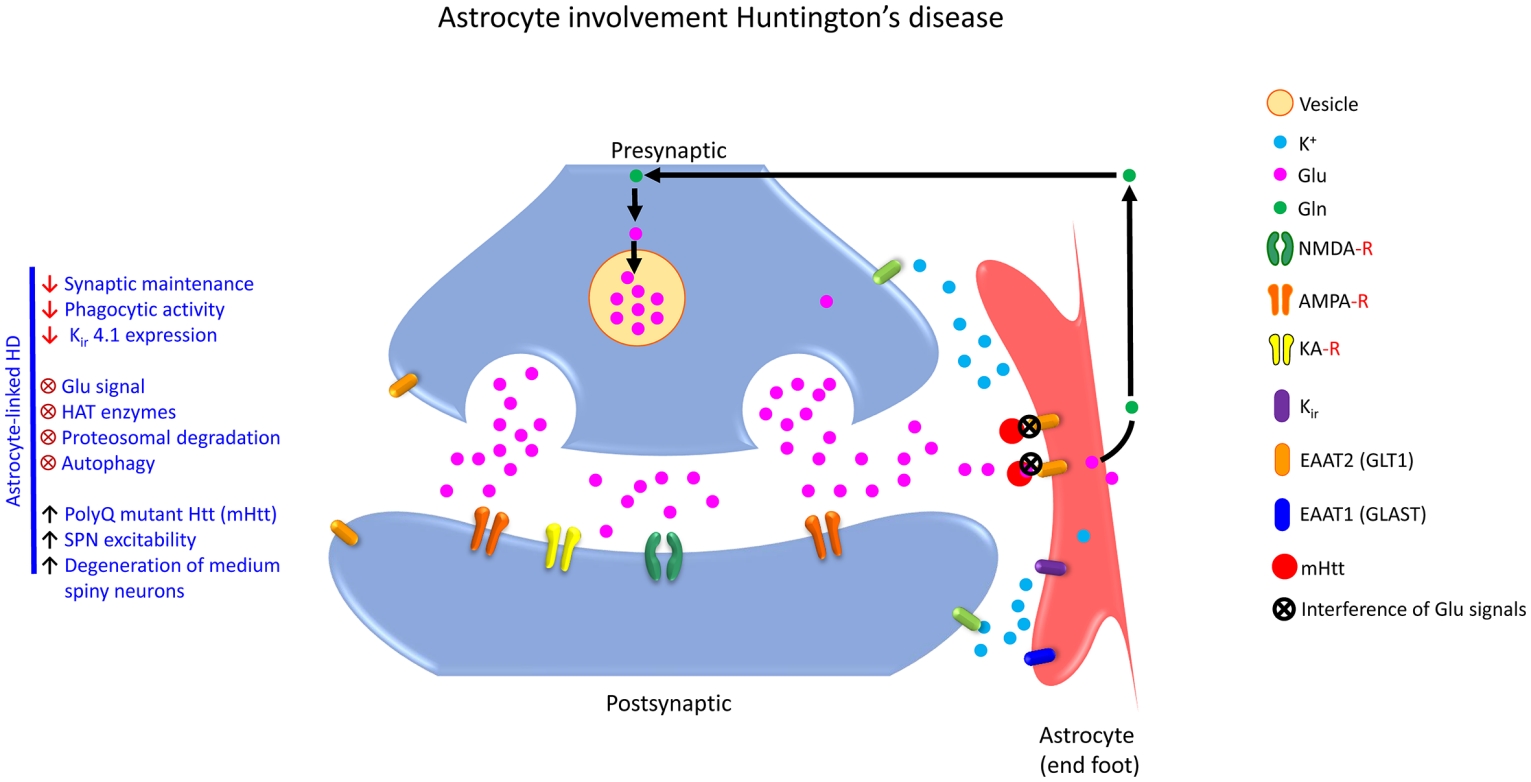
Schematic showing astrocyte involvement in the tripartite glutamatergic synapse in HD. The trinucleotide repeat CAG in the gene mHTT interferes with the Glu signaling cascade because of the abundant presence of mHTT protein in the astrocytes. Here, the Glu uptake function is reduced in the astrocytes due to mHTT aggregation that ultimately impairs feed-forward mechanisms between astrocytes and synapses. The associated pathological features linked to PolyQ mutant HTT (mHTT) are increment in SPN excitability, the high propensity of degeneration of medium spiny neurons and decrement of synaptic maintenance, phagocytotic activity along with Kir_4.1_ expression resulting in the perturbations in growth factor, ion channels, receptors at the molecular and cellular level. K^+^, Potassium ion; Glu, glutamic acid (glutamate); Gln, glutamine; NMDA-R, *N*-methyl-d-aspartate receptor; AMPA-R, 2-amino-3-(3-hydroxy-5-methyl-isoxazol-4-yl) propanoic acid receptor; KA-R, kainate receptor; K_ir_, the inwardly rectifying potassium channels; EAAT2 (GLT1), excitatory amino acid transporter 2 (glutamate transporter 1); EAAT1 (GLAST), excitatory amino acid transporter 1 (glutamate aspartate transporter); mHTT, mutant huntingtin protein.

Huntington’s disease (HD) is an autosomal dominant neurodegenerative disorder that exhibits different characteristic features such as impairment of motor function, cognitive decline, dementia, and death. An expanded chain of polyglutamine in the huntingtin protein (HTT) is the hallmark of HD (Khakh and Sofroniew, 2015), where aggregation and accumulation of intracellular mutant HTT (mHTT) occurs. Using mouse models of HD, it has been shown that the onset and progression of the pathology are associated with astrocyte dysfunction selectively in the striatum, indicating the involvement of specific neural pathways, as well as pathway-specific roles for astrocytes (Khakh and Sofroniew, 2015; Lee et al., 2022). There is also evidence that Kir_4.1_ potassium channels, expressed mainly by astrocytes, are involved in the regulation of extracellular K^+^ levels in the CNS (see Figure 7). Indeed, the loss of Kir_4.1_ currents in striatal astrocytes from HD mouse models has been shown to exhibit higher ambient K^+^ concentrations, which enhances the excitability of the medium spiny neurons (Tong et al., 2014; Khakh and Sofroniew, 2015; Ohno et al., 2021; Lee et al., 2022). Other studies have focused on the loss of Glu transporter function GLT-1 (EAAT2) in the context of HD. In addition, it is well-accepted that mHTT impairs the glutamate uptake in synapses and disrupts the homeostasis of K^+^ in striatal astrocytes from HD mice (Khakh and Sofroniew, 2015; Caron et al., 2018; Lee et al., 2022). Together, mHTT can interfere with Glu uptake and aggregation in the intracellular compartment.

While astrocyte Ca^2+^ dynamics and network response become aberrant in mouse HD models (Jiang et al., 2016), this may lead to cognitive impairment and neuropathology. A recent transcriptomic study revealed that a particular subset or the mixed population of A1-A2 or pan-reactive transcripts are upregulated in astrocytes from the brains of patients with HD (Al-Dalahmah et al., 2020; Lee et al., 2022). As in familial neurodegenerative disorders such as HD, mutations in ubiquitously expressed genes or disease-risk polymorphism in genes highly expressed in astrocytes may also contribute to dysfunctional astrocytes and, consequently adverse neurological outcomes (Lin et al., 2018). The loss of function or alteration in reactive astrocytes not only affects the ion buffering system but also equally affects the neurotransmitter signaling cascade in the animal model of HD (Tong et al., 2014). In HD, a compromised function of astrocytes can elicit a variety of cellular mechanisms, including transcriptional dysregulation, impairment of mitochondrial function, synaptic dysfunction, endoplasmic reticulum (ER) stress, and reduced levels of trophic factors, which cause pathological injury to the medium spiny neurons (Caron et al., 2018; Lee et al., 2022).

#### 4.4.5. Astrocytes and multiple sclerosis

Some of the pathological features noted in multiple sclerosis (MS) constitute demyelination, failure of remyelination, reactive gliosis, oligodendrocyte loss, axonal degeneration, multifocal inflammation, and breakdown of the BBB (Trapp and Nave, 2008; Liddelow et al., 2017). It is now accepted that during demyelination, the energy demand increases for the conduction of nerve impulses. In chronic demyelination, axoplasmic ATP production can become compromised. This type of regular demyelination results in ionic imbalance with a propensity toward axoplasmic Ca^2+^ rise, which causes progressive destruction of axons. The myelination phenomenon has been shown to align with the voltage-gated Na^+^ channels at nodes of Ranvier, which are small unmyelinated axonal segments that separate individual myelin internodes (Trapp and Kidd, 2004; Trapp and Nave, 2008). As soon as demyelination occurs, Na^+^ channels distribute along the entire length of the demyelinated axons (Felts et al., 1997; Trapp and Nave, 2008).

A recent study showed that astrocytes in multiple sclerosis exhibited decreased expression of nuclear factor, erythroid 2 like 2 (also known as nuclear factor, erythroid 2 like BZIP transcription factor 2; NFE2L2/NRF2) and increased expression of V-Maf musculoaponeurotic fibrosarcoma oncogene homolog G (also known as MAF BZIP transcription factor G; MAFG), which cooperate with methionine adenosyltransferase II, alpha (also known as methionine adenosyl transferase 2A; MAT2A/MAT2α) to promote DNA methylation and repression of antioxidant and anti-inflammatory transcriptional signals (Wheeler et al., 2020; Ohno et al., 2021). In the same study, the authors reported that colony-stimulating factor 2 (also known as granulocyte-macrophage colony-stimulating factor; CSF2/GM-CSF) signaling in astrocytes drives the expression of MAFG, MAT2A, and pro-inflammatory transcriptional modules, possibly leading to MS (Wheeler et al., 2020; Ohno et al., 2021). Another study reported that the neurotoxic subpopulation of astrocytes (referred to as A1 astrocytes; Liddelow et al., 2017) is primarily attributed to the activation of microglia for secretion of interleukin 1 alpha (IL1A/IL1α), tumor necrosis factor (also known as tumor necrosis factor-alpha; TNFA/TNFα) and complement component 1, Q subcomponent, A chain (also known as complement C1q A chain; C1QA/C1q), whereas plausible protective mechanisms are attained by a subpopulation of astrocytes (referred as A2 astrocytes) and are attributed to the upregulation of neurotrophic factors.

#### 4.4.6. Astrocytes and amyotrophic lateral sclerosis/motor neuron diseases

Motor neurons are damaged by oxidative stress and reactive astrogliosis in ALS patients (Rao and Weiss, 2004; Iskusnykh et al., 2022). ALS is characterized by the progressive degeneration of upper motor neurons in the cortex and the lower motor neurons in the brainstem and spinal cord (Maragakis and Rothstein, 2006; Brown and Al-Chalabi, 2017; Henstridge et al., 2018). Most ALS cases are sporadic, but about 10% are of familial origin, and ~20% of these cases are linked to mutations in the superoxide dismutase gene (SOD; Rao and Weiss, 2004). Mutant superoxide dismutase 1 (Cu/Zn superoxide dismutase; SOD1) frequently forms intracellular aggregates. Some of these aggregates are detected only in specific ALS subtypes (for example, dipeptide aggregates and intranuclear RNA deposits in *C90RF72* ALS; Brown and Al-Chalabi, 2017). ALS typically begins with focal weakness, which gradually spreads into most of the muscles, including the diaphragm, resulting in death due to respiratory paralysis within 3 to 5 years. SOD1 is increased in the astrocytes of patients with ALS, emphasizing a critical role for astrocytes in the pathogenesis of ALS (Brown and Al-Chalabi, 2017).

Broadhead et al. (2022) reported that large complex post-synaptic structures disappeared in ALS mice making tripartite synapses vulnerable to insult, while non-tripartite synapses remained equal to healthy controls. This led the authors to hypothesize that the synaptic and astrocytic dysfunction could be mechanistically linked and that the tripartite synapse could be pivotal in switching the pathogenesis toward ALS (Broadhead et al., 2022).

It has been reported that mislocalization and aggregation of TAR DNA-binding protein-43 (also known as TAR DNA binding protein; TARDBP/TDP43) from the nucleus to the cytoplasm of neural cells (both neurons and glia) correlates with the loss of synapses in ALS, suggesting a key role for tripartite synapses in the neurodegenerative process (Henstridge et al., 2018). Interestingly, the same authors also discovered phosphorylated TDP43 (pTDP43) puncta in a subset of brain synapses in the prefrontal cortex of ALS patients, suggestive of their involvement in cognitive decline. Thus, the scientific community has concluded that the mislocalization of TDP43 in the cytosol happens and cleavage, phosphorylation, and ubiquitination are some of the exciting features illuminated in sporadic ALS and frontotemporal dementia (Brown and Al-Chalabi, 2017). In ALS pathogenesis, excitotoxic and oxidative crosstalk exists between motor neurons and glia. A feedforward cycle in which neuron-induced chemicals, including oxidants, decrease Glu uptake leading to augmentation of excitotoxic damage, thereby contributing to the progression of neurodegenerative disease (Rao and Weiss, 2004). Mutations in several other genes have also been identified in familial ALS (Maragakis and Rothstein, 2006), which include dynactin subunit I (also known as dynein-associated polypeptide; DCTN1), alsin Rho guanine nucleotide exchange factor ALS2 (also known as amyotrophic lateral sclerosis 2 protein; ALS2), and syntaxin 1A (STX1A). Furthermore, it has been shown that the loss of 95% of astroglial EAAT2 protein expression could be due to the production of truncated EAAT2 and the retention of normal EAAT2 protein in the cytoplasm (Maragakis and Rothstein, 2006).

## 5. Interactions of astrocytes and endothelial system

**Figure 8:**
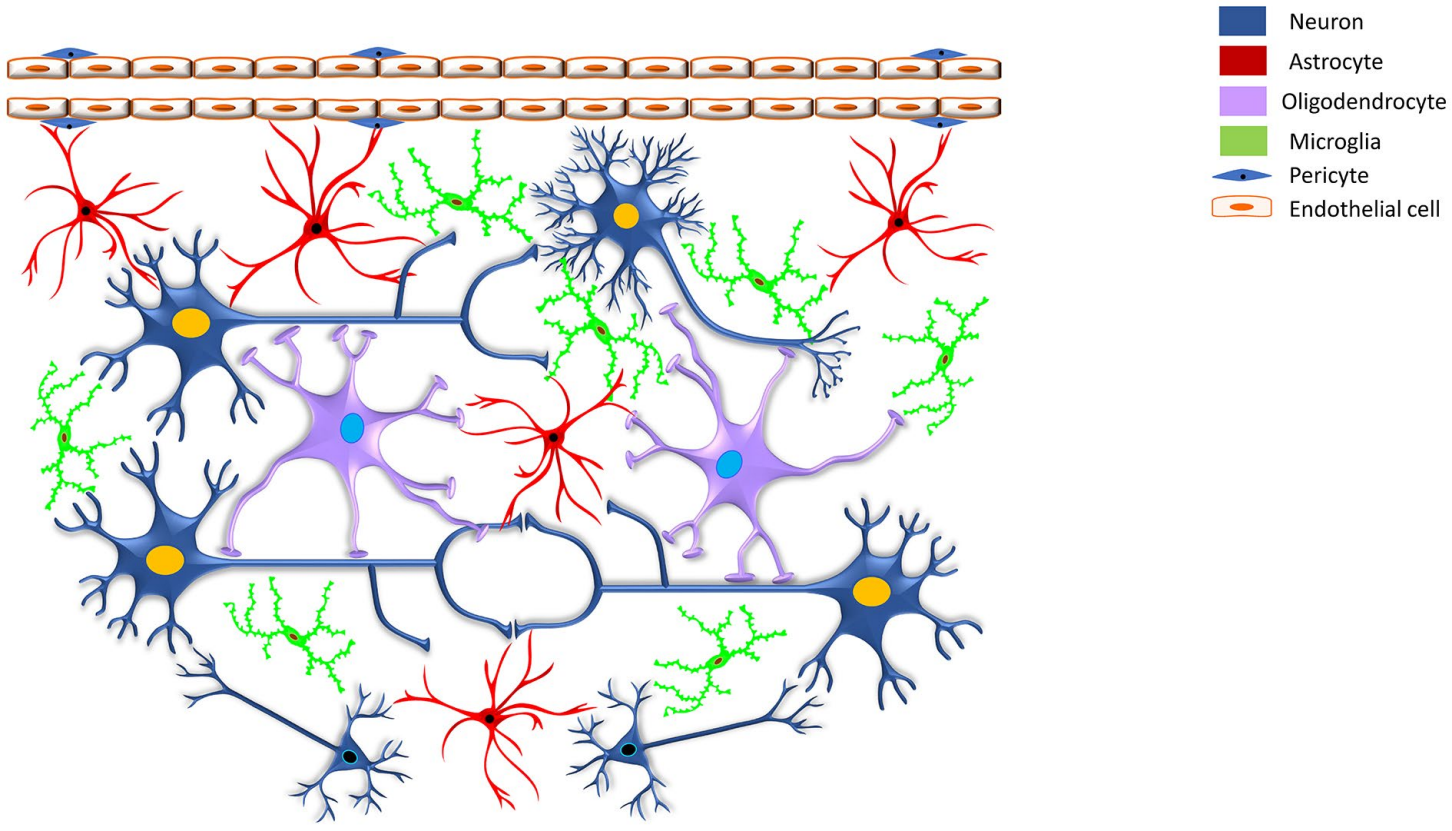
Schematic showing the omnidirectional connections of astrocytes with neurons. Capillary endothelial cells form a blood–brain barrier, surrounded by basal lamina, pericytes, and astrocytic perivascular process. The schematic reveals that the astrocyte end feet are making omnidirectional connections with endothelial cells, neurons, astrocytes, oligodendrocytes, and microglia. To maintain BBB, bidirectional astroglial-endothelial induction is critical, eventually modulating the signaling locally and remotely. At the capillary, synaptic activation triggers ATP, which binds to the astrocyte membrane ion channel P2XR1, increasing intracellular calcium and activating the PGE2 signaling pathway.

In the past two decades, mounting evidence has revealed that astrocytes have a more dynamic function in cerebrovascular regulation (Alvarez et al., 2011; Senatorov et al., 2019; Corkrum et al., 2020). Astrocytic processes elongate their end feet to contact the brain vasculature, which also envelops neuronal synapses (Araya et al., 2008; Salman et al., 2022). The human brain vasculature is extremely complex and has an intricate network of blood vessels and capillaries measuring up to 400 miles. This is critical to supply the brain cells with oxygen, energy, metabolites, and nutrients, as well as remove carbon dioxide and other metabolic wastes from the brain to the systemic circulation (Sweeney et al., 2018; Lee et al., 2022). The inter-relationship between neurons, astrocytes, and blood capillaries, makes the astrocytes a key player and central element in modulating neuronal activity and cerebral blood flow (Sweeney et al., 2018; Lee et al., 2022; see Figure 8). The neuronal guidance cue, Reelin (RELN), demonstrates pro-angiogenic activities and ensures that the communication between the endothelial cells and the astrocytes is intact to control neuronal migration and establishment of the BBB (Segarra et al., 2018). Capillaries are composed of endothelial cells (ECs), which are linked with all arteries and veins (Longden et al., 2021). Astrocytes have been recognized to play a significant role in BBB function by releasing soluble factors to generate a BBB phenotype and other cellular properties in the endothelial system (Alvarez et al., 2011; Reemst et al., 2016). It has been shown that astrocytes secrete sonic hedgehog signaling molecule (SHH) and that the ECs express hedgehog receptors (HH), which collectively promote BBB formation and integrity during embryonic development and adulthood (Alvarez et al., 2011).

A role for astrocytes in regulating BBB properties (Araya et al., 2008) of cerebral endothelium in adult mice through specific bone morphogenetic protein (BMP) signaling mechanisms on astrocyte end-feet has been suggested (Araya et al., 2008), which, when disrupted causes BBB leakiness (Araya et al., 2008). It has also been reported that astrocytes orchestrate responses to cerebral inflammatory diseases, impaired glymphatic function, and other types of CNS injury (Salman et al., 2022). For example, BBB disruption is a central and early warning feature of MS that permits leukocytes to enter the CNS (Alvarez et al., 2011), leading to demyelination and neuronal damage (Ransohoff et al., 2003; Alvarez et al., 2011). Modulating the gain-of-function and loss-of-function (Senatorov et al., 2019; Ohno et al., 2021) in astrocytes revealed that the dysfunction of the BBB initiates hyperactivation of transforming growth factor beta (TGFB/TGF-β) signaling, which is vital to elicit neuronal dysfunction and age-related neuropathology (Senatorov et al., 2019; Ohno et al., 2021). Intriguingly, the factors regulating the brain-immune system communication in healthy conditions are highly complex and incompletely understood. Still, studies have demonstrated that CX43 gap junction proteins are highly expressed in the astrocytes at the BBB interface and are considered an immunoregulating factor (Boulay et al., 2016). Although non-neural, blood-borne cell types may also be involved in neuronal and glial response to injury caused by known dopaminergic toxicant, MPTP, due to their entry *via* a breached BBB, systemically administered MPTP does not compromise the BBB (Sriram et al., 2004). Therefore, astrocytes have emerged as a key component of the CNS vascular system, where they regulate the transfer of metabolites from the blood, communicate with the peripheral immune system, maintain the integrity of BBB, regulate homeostasis, and initiate blood vessel contractility phenomenon upon a neuronal activity (Cohen-Salmon et al., 2021).

One of the critical functions of the astrocyte is buffering of extracellular K^+^, which is essential to ensure normal neuronal excitability (Maragakis and Rothstein, 2006; Sofroniew and Vinters, 2010). Several mechano-transducing ion channels, stretch-sensitive ion channels including big potassium channel alpha subunit (also known as potassium calcium-activated channel subfamily M alpha 1; KCNMA1/BK) channels, are expressed on astrocytes which may contribute to the rapid influx of extracellular Ca^2+^ and Na^+^ from stressed astrocytes in association with NMDA receptors present on the synapses (Burda et al., 2016).

Aquaporin channels assist the bi-directional flow of water and small uncharged solutes, whose membrane permeability is controlled by aquaporin abundance (Salman et al., 2022). The astrocyte endfeet are enriched with aquaporin channels, which mediate water exchange across the blood-spinal cord barrier and the BBB, control cell and extracellular space volume, and astrocyte migration (Salman et al., 2022). To perform the above functions, the cerebrovascular coupling is essential. In cerebral ischemia, a decrease in the blood flow leads to a loss of oxygen and glucose supply to the brain, which results in the loss of the cell’s ability to synthesize ATP and execute oxidative phosphorylation (Burda et al., 2016; Segarra et al., 2018). These conditions are the culprits for excitotoxicity and malfunctioning of the astrocytic GLT1. The fundamental process of clearance of Glu from the synaptic cleft gets hindered in such disease conditions.

*In vitro* and *in vivo* studies using brain cortical tissues have reported that the synaptic release of Glu activates metabotropic Glu receptors on astrocytes, which trigger the arachidonic acid pathway (Lee et al., 2022), resulting in a local increase of Ca^2+^ at astrocyte end-feet, and ultimately leads to the dilation of nearby arterioles (Maragakis and Rothstein, 2006; Lee et al., 2022). Another *in vivo* imaging study reports that brain capillary endothelial cells control blood flow through a hierarchy (Longden et al., 2021) of inositol 1,4,5-triphosphate receptor, type 3 (IPTR3)-mediated Ca^2+^ events, ranging from small, sub-second proto-events, demonstrating Ca^2+^ release through a small number of channels, to high amplitude, sustained (up to ~1 min) compound events mediated by large clusters of channels (Longden et al., 2021; Jackson, 2022). Longden et al. (2021) also interpreted the mechanism of how neuronal activity drives Ca^2+^ signal, engages Gq protein-coupled receptor (GPCR) signaling, and is augmented by Ca^2+^ entering *via* transient receptor potential cation channel subfamily V member 4 (also known as vanilloid receptor-like channel 2; TRPV4) channels.

## 6. Astrocyte senescence and neurological dysfunction

A time-dependent decline or degradation of the molecular, cellular, and physiological functions of living entities is characteristic of aging. Aging is the most important risk factor associated with neurodegenerative diseases, like AD and PD, which are thought to be due to the accumulation of senescent astrocytes (Verkerke et al., 2021; Preininger and Kaufer, 2022). Senescent astrocytes display a variety of changes with normal aging, including the reduced function of the antioxidant glutathione system, decreased antioxidant function, decreased conversion of glutamate to Gln as a consequence of a decline in GLUL enzyme activity, reduced clearance of Aβ, altered cholesterol metabolism, shortening of telomeres and telomere attrition with age, excessive accumulation of EAAT1, enhanced expression of matrisome-related molecules, increased oxidative phosphorylation (OxPhos), reduced efficiency of maintaining neurotransmitter homeostasis, reduced astrocytic interaction with neighboring cells, impaired lipid transport, increased complement levels, increased lipid peroxidation, and a heightened inflammatory state (Palmer and Ousman, 2018; Cohen and Torres, 2019; Fernandez et al., 2019; Verkerke et al., 2021). Aged astrocytes are also morphologically and transcriptionally different from young astrocytes indicating that age-related functional changes occur in astrocytes (Verkerke et al., 2021). Specifically, the long and slender astrocyte phenotype changes to a short stubby structure, and the astrocyte endfeet retracts from the endothelium (Palmer and Ousman, 2018; Kalaria and Hase, 2019). Further, there is evidence that age-associated BBB dysfunction causes hyperactivation of TGFB signaling in astrocytes, which elicits a proinflammatory and epileptogenic phenotype, thereby downregulating aquaporin 4 (AQP4) channels, glutamate transporters (EAAT1/2) and K_ir_4.1 (Preininger and Kaufer, 2022). Collectively, these studies provide evidence for the potential role of astrocyte senescence and dysfunction in eliciting neuroinflammation, neurotoxicity, neuronal hyperexcitability, and neurodegeneration in the aging brain.

## 7. Functional implications

The past two decades of investigations have seen an immense increase in the knowledge about the communication of astrocytes in the brain. There is also a substantial accumulation of experimental data demonstrating the active role of astrocytes in the physiology and pathophysiology of the CNS. It is now high time to interpret these data in such a way as to assemble a collection of meaningful and testable hypotheses about the mechanisms of astrocyte signaling, addressing both synaptic and non-synaptic processes. Studies that selectively examine excessive neurotransmitter release, and interaction with astrocytes, synapses, and ventricular sites, will be highly insightful and can provide the foundation to unravel other unknown mechanisms related to the role of astrocytes in neurological and neurodegenerative diseases. The limitation persists, first, by the complexity and functional diversity of the projection of the astrocytes in the CNS and, second, by the multitude of functions neurotransmitters exhibit, including excitotoxicity and depressive-like events. Many of these have properties well beyond those involved in maintaining homeostasis. The lack of technology to apply electrophysiology without labeling astrocytes has added another layer of difficulty in assessing the role of each astrocyte in health and disease. Many studies use antibody-mediated blockade or gene-deletion approach, but the results are inconsistent. It is, therefore, imperative to design and test ingenious strategies to examine each astrocyte function on inhibitory and excitatory synapse, microglia, and vasculature to probe into the disease mechanisms for accurate therapeutic intervention. We anticipate that, along with technological advances, novel molecular and cellular links will be revealed. New interventions will be developed to be applied across a wide range of neurological and neurodegenerative disorders affected by astrocytic regulation. In the coming years, researchers will shed further light on the impact of astrocytes on neuronal function in health and disease, and perhaps time will tell these cardinal directions.

## 8. Conclusion

From the foregoing discussion, the omnidirectional communication of astrocytes and neurons at the tripartite synapse exists. Since astrocytes are closely linked with the synapse, which receives signals from the pre-synaptic neuron and responds by releasing feedback signals, they play a crucial role in brain function in health and disease. Mechanistic insight into the link between astrocytic regulation and illness can be leveraged to identify novel strategies of therapies and new treatment targets. Distinct mediators released by subtypes of astrocytes could be valuable biomarkers in detecting neurological and neurodegenerative disorders. The chemical cue for synaptic function, axonal guidance, and axonal outgrowth needs further elaboration in the presence of astrocytes. In this review, we have made a zealous attempt to sufficiently identify and provide substantial facts to probe into the dynamic role of astrocytes in health and disease by compiling investigations related to the underlying mechanisms.

## Author contributions

KS and DP conceived and designed the review article. DP conducted the literature search and drafted the manuscript. KS prepared the tables, illustrations, and revised the manuscript. All authors have read and agreed to the published version of the manuscript.

## Literature selection criteria

*Inclusion criteria*:

PubMed, including MEDLINE and PubMed Central were the primary databases for extensively searching and gathering literature. In addition, several neuroscience journals were also accessed directly. All the full-text literature were selected as relevant without any bias. Further, all studies utilizing in vitro, animal models, or human subjects were included.

*Exclusion criteria*:

The primary criteria for exclusion were (i) articles not in English, (ii) searches that recovered abstracts only, (iii) out of the context articles that diverted from the astrocytic-neuronal perspective.

## Funding

Funding was provided by the National Institute for Occupational Safety and Health through NORA project number 9390BN0 (to KS) and NTRC project number 9390KK9 (to KS).

## Conflict of interest

The authors declare that the research was conducted in the absence of any commercial or financial relationships that could be construed as a potential conflict of interest.

## Publisher’s note

All claims expressed in this article are solely those of the authors and do not necessarily represent those of their affiliated organizations, or those of the publisher, the editors and the reviewers. Any product that may be evaluated in this article, or claim that may be made by its manufacturer, is not guaranteed or endorsed by the publisher.

## Author disclaimer

The findings and conclusions in this report are those of the author(s) and do not necessarily represent the official position of the National Institute for Occupational Safety and Health, Centers for Disease Control and Prevention.
